# Ambient Air Pollution and Respiratory Health in Sub-Saharan African Children: A Cross-Sectional Analysis

**DOI:** 10.3390/ijerph18189729

**Published:** 2021-09-15

**Authors:** Yutong Samuel Cai, Harry Gibson, Rema Ramakrishnan, Mohammad Mamouei, Kazem Rahimi

**Affiliations:** 1 Nuffield Department of Women’s & Reproductive Health, University of Oxford, Oxford OX3 9DU, UK; harry.s.gibson+research@gmail.com (H.G.); Mohammad.Mamouei@wrh.ox.ac.uk (M.M.); kazem.rahimi@wrh.ox.ac.uk (K.R.); 2 Deep Medicine Programme, Oxford Martin School, University of Oxford, Oxford OX1 3BD, UK; 3 Informal Cities Programme, Oxford Martin School, University of Oxford, Oxford OX1 3BD, UK; 4 National Perinatal Epidemiology Unit, Nuffield Department of Population Health, University of Oxford, Oxford OX3 7LF, UK; rema.ramakrishnan@npeu.ox.ac.uk

**Keywords:** outdoor air pollution, fine particles, lung health, children, Africa

## Abstract

Ambient air pollution is projected to become a major environmental risk in sub-Saharan Africa (SSA). Research into its health impacts is hindered by limited data. We aimed to investigate the cross-sectional relationship between particulate matter with a diameter ≤ 2.5 μm (PM_2.5_) and prevalence of cough or acute lower respiratory infection (ALRI) among children under five in SSA. Data were collected from 31 Demographic and Health Surveys (DHS) in 21 SSA countries between 2005–2018. Prior-month average PM_2.5_ preceding the survey date was assessed based on satellite measurements and a chemical transport model. Cough and ALRI in the past two weeks were derived from questionnaires. Associations were analysed using conditional logistic regression within each survey cluster, adjusting for child’s age, sex, birth size, household wealth, maternal education, maternal age and month of the interview. Survey-specific odds ratios (ORs) were pooled using random-effect meta-analysis. Included were 368,366 and 109,664 children for the analysis of cough and ALRI, respectively. On average, 20.5% children had reported a cough, 6.4% reported ALRI, and 32% of children lived in urban areas. Prior-month average PM_2.5_ ranged from 8.9 to 64.6 μg/m^3^. Pooling all surveys, no associations were observed with either outcome in the overall populations. Among countries with medium-to-high Human Development Index, positive associations were observed with both cough (pooled OR: 1.022, 95%CI: 0.982–1.064) and ALRI (pooled OR: 1.018, 95%CI: 0.975–1.064) for 1 μg/m^3^ higher of PM_2.5_. This explorative study found no associations between short-term ambient PM_2.5_ and respiratory health among young SSA children, necessitating future analyses using better-defined exposure and health metrics to study this important link.

## 1. Introduction

Air pollution disproportionately affects populations residing in low and middle income countries (LMICs). In 2015, 89% of deaths due to ambient (outdoor) air pollution occurred in LMICs, mainly in Asia and Africa [1]. Africa has the highest excess mortality from ambient air pollution among children under five years of age (under five, 0–59 months), to which acute lower respiratory infection (ALRI) was suggested as a potential contributor [2]. 

ALRI, including infections of both alveoli (e.g., pneumonia) and airways (e.g., bronchiolitis and bronchitis), is the most common cause of illness among young children worldwide; in sub-Saharan Africa (SSA), it is a major cause of death in children under five [3]. While ALRI is caused by infective agents (either bacterial or viral), there is a wide range of risk factors that are associated with it. These factors include child’s age, socio-economic status, underlying health conditions, immunisation, access to health care and household air pollution [4]. 

Associations between long- or short-term ambient air pollution and childhood pneumonia or hospitalisation for ALRI have been consistently reported in North America [5,6], Europe [7] and recently in Asia [8,9,10,11]. Urban particulate matter (PM) and its compositions (e.g., heavy metals) seem to be particularly harmful for acute respiratory events among young children [12,13]. However, there is a dearth of data for such associations in the SSA region [14]. Among the limited number of studies in Africa, most have used crude proxies of ambient air pollution (e.g., residential proximity to industrial areas, heavy-traffic roads or mine dumps) instead of individual air pollutants [15,16]. This has largely explained the inconsistent findings across the studies and also inhibited comparisons with studies in which individual air pollutants were investigated. For most SSA countries, a lack of ambient air quality monitoring, together with inadequate capacity for collecting population health data, has hindered the progress in conducting ambient air pollution research on population health [17]. 

Remote-sensed technology is increasingly used to predict ground-level air pollutant concentrations, providing an opportunity to conduct air pollution health research in the SSA region by linking publicly available health surveys. In this study, we combined standardised data of the Demographic and Health Surveys (DHS) from 21 SSA countries with satellite-derived PM_2.5_ (PM with a diameter less than or equal to 2.5 micrometres) concentrations. We aimed to analyse the cross-sectional associations between short-term exposure to ambient PM_2.5_ and self-reported ALRI or cough among children under five in SSA. 

## 2. Materials and Methods

### 2.1. Data Source and Study Population

DHS collect nationally representative health and sociodemographic data every few years in over 90 countries globally (https://dhsprogram.com/, accessed on 14 September 2021). A two-stage sampling strategy is adopted, in which surveyed clusters are randomly drawn from census files and then households are randomly selected within each surveyed cluster. Data are collected from all members in each selected household via standardised questionnaires and physical examinations (i.e., anthropometric data) by trained field staff. Standard DHS surveys conducted between 2000s and 2018 with available, and valid geolocations of the survey clusters were included in this study. This study focused on children under five, who are either a member of the surveyed household or stayed overnight at the household before the staff’s visit. DHS survey protocols have been ethically reviewed and informed consent was obtained orally from participants. 

### 2.2. Outcomes and Covariates

As with a previous study [18], outcomes were derived from answers to the two questionnaire items reported by mothers or other caregivers: (1) “*Has the child had an illness with a cough at any time in the last two weeks*?” and (2) “*Has the child had fast, short, rapid breaths or difficulty breathing at any time in the last two weeks*?” ALRI was defined if answers were positive to both questions, whilst cough was defined if the answer was positive to the first question only. 

Based on the current knowledge [4] and data availability, covariates to be adjusted for in the statistical analyses were selected a priori. These include child’s sex, child’s age in months, size at birth (very large, larger than average, average, smaller than average, and very small), calendar month of the interview, household wealth index, maternal age, maternal education (no formal education, primary school level, and secondary school or above), ownership of a health card (yes/no), child stunting (yes/no) and types of cooking fuel (electricity/gas vs. wood/coal/others). Most of all these covariates were directly obtained through the questionnaire. Household wealth index (poorest, poorer, average, wealthier, wealthiest) was subsequently constructed in quintiles by DHS through a principal component analysis based on collected data on household asset ownership, access to utilities and housing construction materials [19]. Stunting status was defined by DHS as height-for-age *z-score* two or more standard deviations below the reference median.

### 2.3. Ambient PM_2.5_ Data

A state-of-the-art global annual surface PM_2.5_ concentration for 1998–2018 (V4.GL.03) was estimated by the Atmospheric Composition Analysis Group. The data are publicly available at http://fizz.phys.dal.ca/~atmos/martin/?page_id=140#V4.GL.03 (accessed on 14 September 2021) [20]. Based on the satellite-retrieved aerosol optical depth (AOD), surface concentrations of PM_2.5_ were estimated using geophysical relationships between AOD and surface PM_2.5_ in a chemical transport model. The estimated surface concentrations of PM_2.5_ were subsequently calibrated globally via Geographically Weighted Regression (GWR) using ground-level monitored PM_2.5_ datasets compiled by the World Health Organization (WHO). The predicted annual surface PM_2.5_ concentrations were in a resolution of 0.01° × 0.01° (~1.11 km) and highly consistent with monitored PM_2.5_ around the globe (*r* = 0.92, year 2015).

To better temporally align ambient PM_2.5_ exposure with the self-reported respiratory outcomes in the past two weeks from the interview date of the DHS survey, we downscaled the aforementioned annual surface PM_2.5_ to a monthly surface. To enable this conversion, we used monthly concentrations of PM_2.5_ in a resolution of 0.5° × 0.625° from the Modern-Era Retrospective Analysis for Research and Applications, version 2 (MERRA-2) [21], freely provided by the United States National Aeronautics and Space Administration. Details of the conversion are described in Supplementary Text S1.

For every DHS surveyed household, based on their aggregated survey cluster’s latitude/longitude and the interview date, we assigned average PM_2.5_ estimates for each of the 12 months preceding the interview. The 15th of the month was used as the reference date for allocation of household to the reference month. For example, any household interviewed between the 15 July 2001 and 14 August 2001 (inclusive) would be counted as “July 2001” and would receive average PM_2.5_ data of July 2001 for month 0 (i.e., Lag0, concurrent month), June 2001 for month 1 (i.e., Lag1, previous month), through to August 2000 for month 12 (i.e., Lag12).

### 2.4. Statistical Analyses 

As with previous studies [18,22], a survey was excluded from the analysis if missingness was greater than 10% for any variable including the study outcomes, PM_2.5_ exposure and covariates adjusted in the main model. For each included survey, the association between PM_2.5_ concentration and the odds of prevalent cough or ALRI was modelled, adjusting for an a priori set of main covariates. Consistent with the DHS survey design, a conditional logistic regression model was applied with consideration of sampling weights in each survey. Associations were only to be estimated within the same geographic survey cluster, an approach minimising inter-cluster confounding factors such as healthcare resources.

The main exposure of interest was average PM_2.5_ concentration in the previous month (i.e., Lag1_PM_2.5_) prior to the DHS survey. The main model was adjusted for child’s sex, child’s age in months, size at birth, month of the interview, household wealth index, maternal age, and maternal education. Month of the interview was included to account for local seasonality of both air quality and respiratory symptoms. Based on the main model, three sensitivity analyses were performed. First, ownership of a health card, as a proxy of vaccination records, was further adjusted. Second, type of cooking fuel, which is generally seen as an indicator of household air pollution, was further adjusted. Third, child stunting as a marker of long-term malnutrition was further adjusted.

Subgroup analyses were conducted by factors that are expected to impact respiratory symptoms and/or PM_2.5_ exposure; these factors include urban/rural cluster, sex, age (0–24 months vs. 25–59 months), stunting status, household wealth index (poorer/poorest versus average and above), west Africa/rest of Africa, and the surveyed country’s human development index (HDI) (medium-to-high (HDI ≥ 0.55 vs. low (HDI < 0.55)). HDI is an index that measures three key dimensions (life expectancy, access to education and standard of living) in every country of the world and is updated on a yearly basis. For this study, HDI data for each respective survey year in each country were downloaded from a public database [23].

The investigated associations in each included survey were individually analysed and then the survey-specific odd ratios (OR) were pooled via random-effect meta-analyses with the DerSimonian and Laird method [24]. The heterogeneity of the survey-specific ORs was assessed using the I^2^ index and *p*-value. Analyses were conducted in Stata 16 (StataCorp, College Station, TX, USA).

## 3. Results

Data from 31 standard DHS surveys conducted in 21 SSA countries were included in this study. Complete data of the outcome, PM_2.5_ exposure and variables in the main model were available for 368,366 and 109,664 children for the analysis of cough and ALRI, respectively.

Across the 31 surveys, prevalence of cough ranged from 7.1% to 39.3%, with an average of 20.5% (Table 1). Eighteen surveys were excluded from the analysis on ALRI due to missing data which was over 10% in each survey. In the remaining 13 surveys, prevalence of ALRI ranged from 2.1% to 14.4%, with an average of 6.4%. On average, 32.4% of the surveyed clusters were in urban areas, 16.8% children had smaller birth size than average, 43.6% of children came from a household with a wealth index below the average of all surveyed households, and 35.8% of mothers had never received any formal education.

Mean PM_2.5_ concentration in the month preceding the interview date (e.g., Lag1) ranged from 6.4 to 64.6 μg/m^3^ while annual mean PM_2.5_ concentration across the survey clusters ranged from 8.5 to 78.8 μg/m^3^ (Table 2). West SSA countries (Burkina Faso, Cameroon, Ghana, Guinea, Mali, Nigeria, Senegal, Togo) had a higher mean PM_2.5_ concentration than the rest of SSA (40.8 vs. 17.1 μg/m^3^ for previous-month mean PM_2.5_ while 53.4 vs. 16.9 μg/m^3^ for annual mean PM_2.5_).

Pooling all individual surveys, no associations were observed between previous-month mean PM_2.5_ concentration and either prevalence of cough (pooled OR: 1.000, 95%CI: 0.981–1.009) or ALRI (pooled OR: 0.975, 95%CI: 0.941–1.010) in the overall population (Table 3), with significant heterogeneity observed among surveys. Sensitivity analyses did not substantially change the main results (Supplementary Table S1). Similarly, no associations were seen for any subgroup analyses by pooling all individual surveys. For the prevalence of cough, positive associations were seen among children from poorer households, from west African countries or from the countries with medium-to-high HDI index. While for the prevalence of ALRI, the association was positive (pooled OR: 1.018, 95%CI: 0.975–1.064) by pooling surveys with a medium-to-high HDI index.

Across each individual survey, a few statistically significant positive associations with cough were seen (e.g., Zimbabwe 2005, Burkina Faso 2010, Ghana 2014, Angola 2016) in the overall population, with the OR ranging from 1.016 to 1.130 per each μg/m^3^ higher of PM_2.5_ (Figure 1). All these associations, except for Ghana 2014, were only found to be significantly positive among urban clusters. In contrast, for Ghana 2014, a positive association was only found among rural clusters (pooled OR: 1.178, 95%CI: 1.129–1.229). Figures of survey-specific ORs for each association are listed in Supplementary Files (Figures S1–S29).

## 4. Discussion

To our knowledge, this is the first multi-country study investigating the associations between short-term ambient PM_2.5_ exposure and respiratory health in children under five across sub-Saharan Africa. Whilst we hypothesised that higher ambient PM_2.5_ exposure is associated with higher odds of cough or ALRI among children under five, our pooled analyses were unable to provide such evidence. Significant heterogeneity was observed for most pooled associations, highlighting the complexity in studying health impacts of air pollution in a vast geographical region such as SAA with a diverse demographic and socioeconomic context. Nonetheless, in some individual surveys, the association with cough was positive and reached statistical significance.

The main strengths of our study include multi-country national surveys from 21 countries across SSA, pooled analyses of individual national surveys with a harmonised set of major confounders at both household- and child-level, and adoption of a standardised assessment of PM_2.5_ exposure which was estimated monthly prior to survey date.

This study has several key limitations that may in part explain the observed null associations. First and foremost, self-reported data on cough and ALRI were non-specific and subject to recall bias. This potential recall bias has led to a misclassification of outcomes and/or masking of associations with more severe infections, thereby reducing the analysis strength in each individual survey. It would be a highly valuable advantage if ALRI could be laboratory-confirmed or clinician-diagnosed, but adoption of which would inevitably preclude studies in many SSA countries as most still do not have adequate access to diagnostic tools or formal healthcare [25]. Second, uncertainties of the global PM_2.5_ model for the SSA region are likely high, as very few ground-level monitored PM_2.5_ data was available in this region to validate the model [20]. In addition, the extent of exposure misclassification for rural clusters are relatively larger as compared to urban clusters. The chemical transport model in estimating PM_2.5_ exposure was based on emission inventories, which have a higher degree of uncertainty in rural areas where biomass and crop residual burning is highly prevalent in rural SSA [26]. This global PM_2.5_ model may also not capture emissions associated with different types of cooking stoves or ventilations in most surveyed households in our study. In our analysis, 68% of surveyed clusters were classified as rural, therefore the uncertainty in exposure assessment is expected to be much higher and may in part lead to the null findings. PM_2.5_ estimates were only assigned to each child at cluster locations, coordinates of which were displaced by 2 to 10 km by DHS to protect participants’ confidentiality. Without individual residential-based PM_2.5_ estimates, together with time-activity data, a larger extent of exposure misclassification is expected to attenuate associations towards null. Third, although our associations have been adjusted for several major confounders, and robustly tested in different sensitivity analyses, it is always probable there exists residual confounding. For instance, we did not have data on comorbidities (HIV, malaria, etc.) that may increase a child’s vulnerability to adverse effects of air pollution. We either did not have data on temperature and humidity which are likely to affect both short-term air pollution and respiratory symptoms. Lastly, this cross-sectional study is inherently exploratory and any observed statistically significant associations do not imply causality.

There are several suggested pathways by which PM_2.5_ may lead to respiratory infection. Inhalable PM_2.5_ could serve as a carrier, and promote the growth of infectious agents which increase the infection per se [27]. PM_2.5_ may also increase the host’s susceptibility to respiratory pathogens via inflammation and oxidative stress, which disrupt the lung’s innate immune system including impaired mucociliary clearance, macrophage function and epithelial barrier [28]. Urban PM_2.5_ has been shown to increase adhesion of pneumococci, a pathogen responsible for most bacterial pneumonia cases, into human airway epithelial cells [29]. However, the interaction between respiratory syncytial virus (RSV), a major cause of ALRI among young children, and PM-exposed airway cells remains unclear [30], despite the fact that recent epidemiological evidence indicated an association between PM_2.5_ and clinically proven RSV infection among children under two years of age [5].

Previously, a robust relationship was found between annual PM_2.5_ exposure and infant mortality across SSA countries, with the risk increased by 9.2% with every 10 μg/m^3^ increase in ambient PM_2.5_ in the first 12 months of life [31]. The authors speculated that ALRI could be one of the many mediators between ambient PM_2.5_ and infant mortality, although our analysis did not provide such direct evidence. A very limited number of African studies suggested that traffic- or industry-related air pollution [32,33,34,35,36], which most often occurs in urban areas, was associated with wheeze, cough, allergic rhinitis and phlegm in children. However, most of these studies had only assessed residential proximity to main road, industrial or mine areas rather than individual air pollutants [15,16]. More recently, a study in the Western Cape province found that modelled-NO_2_, but not modelled-PM_2.5_, was significantly associated with a new onset of asthma symptoms among school-aged children residing in informal settlements [37].

Globally, the associations between daily mean PM_2.5_ and childhood pneumonia or ALRI hospitalisation were observed in a wide range of PM_2.5_ exposure (range: 9.4 to 96.0 μg/m^3^), including that below the current WHO guideline (25 μg/m^3^ daily mean) [14]. In an analysis of 112,467 children aged 0–2 years in the Wasatch Front region of Utah (largely urban/suburban), cumulative 28-day PM_2.5_ exposure (daily mean: ~10 μg/m^3^) was significantly associated with clinically verified ALRI (OR: 1.15, 95%CI: 1.11–1.19, per 10 μg/m^3^ increase) [5]. Collectively, all these studies have strengthened the call to reduce ambient air pollution, including PM_2.5_, as much as possible to protect health. In the previous analysis of DHS survey data, it was estimated that 22% infant deaths (~449,000) could be avoided should the annual average of PM_2.5_ was reduced to 2 μg/m^3^ in SSA [31].

Whilst overall our study did not suggest an association between ambient PM_2.5_ and respiratory health in children under five across SSA, it did shed lights into some works that should be urgently addressed. For example, positive associations with higher effect sizes were observed with both cough and ALRI, albeit non-significant, when analysis was restricted to countries with a medium-to-high HDI index. This may highlight the potential harm of urbanisation, which acts partly via decreased air quality on human health. Sub-Saharan Africa is urbanising at an unprecedented rate and is projected to pass the 50% urban tipping point by 2035 [38]. This rapid urbanisation, together with inadequate capacities in policy regulations and supporting infrastructure, are expected to bring unfavourable changes in urban environment, including the air quality [39]. Some African cities already reported that concentrations of air pollutants were higher in urban business and high-density residential areas, where traffic and biomass use are the dominant sources, than in peri-urban or rural areas where traffic is considerably less [40,41,42]. Traffic-related air pollution from both formal and informal transport sectors is becoming a major health concern in urban SSA. Across SSA cities, whilst the overall number of new vehicles is increasing over time, second-hand and diesel-powered vehicles are still imported; much often the vehicle fleet is poorly maintained and emissions standards are either completely lacking or not strictly enforced in most SSA countries [17]. There is already established evidence from other global urban regions that diesel exhaust from traffic is particularly harmful to a range of childhood respiratory diseases [43]. To safeguard public health from air pollution, we echo with other reports that African cities should endeavour to establish and maintain an air quality monitoring network [44], data of which has proven to be extremely important for both policy-making and scientific research.

## 5. Conclusions

By pooling national survey data across SSA countries, this explorative study did not observe an association between short-term ambient PM_2.5_ exposure and respiratory health among young children. Future works are urgently needed in SSA to better characterise concentrations, distributions and toxicity of ambient air pollution, as well as their impacts on the developing lungs in children.

## Figures and Tables

**Figure 1:**
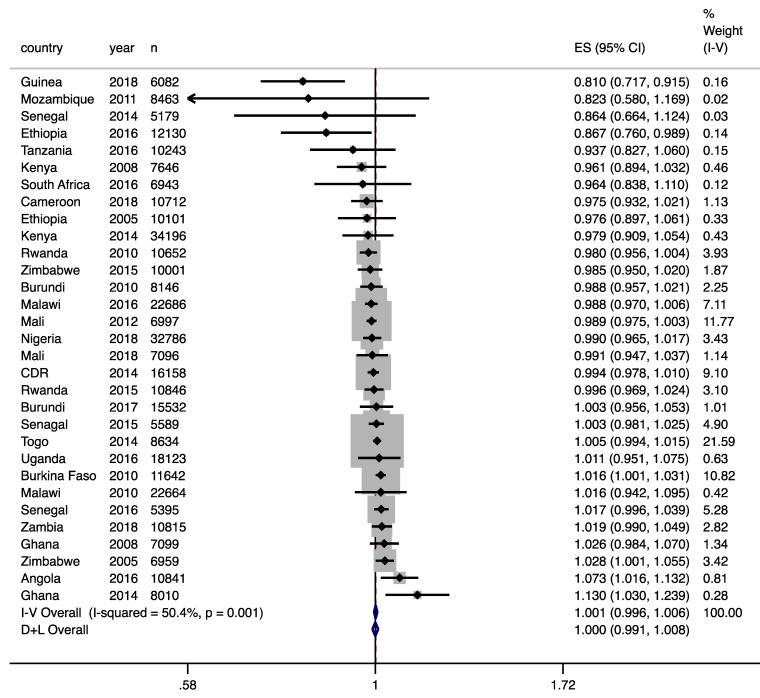
Associations between prior-month PM_2.5_ concentration (per 1 μg/m^3^ higher) and prevalence of cough in the past two weeks across DHS in sub-Saharan Africa.

**Table 1:** Characteristics of study participants.

Country, Year	No. of Under-5 Children	No. of Under-5 Children with Data on Cough	% with Cough	No. of Under-5 Children with Data on ALRI	% with ALRI *	% Urban Clusters	Mean Child’s Age in Months	% Girl	% with Smaller Birth Size than Average *	% Poorer & Poorest Households	Mean Maternal Age in Years	% No. Formal Education
Angola, 2016	17,367	16,409	10.5	16,374	4.1	55.3	29.6	50.7	9.7	47.7	28.0	35.0
Burkina Faso, 2010	17,249	15,628	10.8	1680	-	27.3	42.7	49.5	13.0	37.1	29.3	80.4
Burundi, 2017	16,949	16,074	37.7	16,072	12.2	18.9	30.4	49.7	16.1	43.8	30.5	42.7
Burundi, 2010	9403	8737	37.6	3280	-	19.6	42.8	48.9	16.7	37.5	30.3	47.9
Cameroon, 2018	13,102	12,250	18.6	12,225	3.8	52.4	30.2	50.2	13.5	36.1	28.5	19.8
CDR, 2014	20,309	18,470	30.4	5591	-	29.7	44.0	50.3	12.1	49.0	29.1	21.0
Ethiopia, 2016	18,324	17,353	15.5	17,331	7.2	31.0	30.3	48.4	25.6	49.6	29.2	54.0
Ethiopia, 2005	15,033	13,681	15.1	2048	-	26.5	37.6	48.2	27.5	38.0	28.9	67.9
Ghana, 2014	12,934	12,149	13.8	1615	-	50.2	44.2	48.4	17.6	42.8	30.7	27.9
Ghana, 2008	12,233	11,313	21.4	2420	-	43.9	41.9	51.3	14.7	41.2	30.2	28.7
Guinea, 2018	9170	8361	12.3	8356	4.6	31.3	29.1	48.8	11.1	45.1	29.2	75.5
Kenya, 2014	39,985	38,108	36.3	13,782	-	38.6	44.2	49.7	-	46.3	28.6	16.2
Kenya, 2008	10,174	9486	23.2	2185	-	32.5	42.0	50.0	16.1	35.2	28.6	15.7
Malawi, 2016	27,594	25,933	23.6	25,879	10.1	18.8	30.7	50.7	15.1	44.3	28.0	11.7
Malawi, 2010	27,516	25,189	28.0	7027	-	11.5	42.6	50.6	14.8	43.9	28.3	15.8
Mali, 2018	11,269	10,470	9.0	10,468	3.1	28.6	29.6	48.7	14.6	43.1	28.6	73.2
Mali, 2012	11,723	10,787	8.0	850	-	26.6	43.6	48.7	14.0	38.1	28.4	81.5
Mozambique, 2011	14,510	13,322	10.9	1434	-	35.8	43.1	50.0	13.6	34.5	28.3	32.3
Nigeria, 2018	46,230	42,199	15.5	42,196	4.9	39.0	30.8	49.1	12.5	37.9	29.9	36.2
Rwanda, 2015	12,834	12,213	25.2	3074	-	23.0	44.4	48.0	15.0	44.2	30.2	14.2
Rwanda, 2010	12,897	12,029	23.3	2800	-	15.9	42.8	47.8	16.3	43.3	30.7	18.0
Senegal, 2014	7063	6515	7.1	466	-	31.7	44.2	50.4	32.7	60.1	29.5	70.7
Senegal, 2015	7286	6679	10.9	727	-	29.6	44.6	50.0	31.8	56.7	29.5	69.0
Senegal, 2016	7147	6610	9.6	634	-	30.0	45.0	48.2	30.9	61.0	29.5	67.8
South Africa, 2016	13,792	12,854	25.3	12,800	4.6	60.0	32.0	45.4	13.8	41.5	28.9	1.0
Tanzania, 2016	14,449	13,405	16.7	13,388	4.9	27.9	29.6	49.0	10.2	37.1	29.2	19.2
Togo, 2014	10,384	9626	24.7	2381	-	37.0	44.0	50.1	16.4	41.1	30.2	39.6
Uganda, 2016	21,356	19,957	39.3	19,930	14.4	22.8	30.7	48.9	19.5	42.7	28.7	12.0
Zambia, 2018	13,905	12,983	20.8	12,981	2.1	35.6	29.4	50.0	12.0	46.6	28.4	9.5
Zimbabwe, 2015	11,202	10,512	35.7	10,461	7.8	41.2	30.7	48.5	14.1	44.8	28.6	1.2
Zimbabwe, 2005	10,784	9725	18.2	1763	-	32.0	40.8	50.5	14.2	40.2	27.8	4.0
Average	-	-	20.5	-	6.4	32.4	37.7	49.3	16.3	43.6	29.1	35.8

No., number; ALRI, acute lower respiratory tract infection. * Prevalence was not computed if missingness of data are >10%.

**Table 2:** Distributions of PM_2.5_ concentration (μg/m^3^) across the surveys.

Country, Year	Number of Under-5 Children	Number with Data on PM_2.5_	Previous-Month PM_2.5_			Annual-PM_2.5_			Spearman *R* *
			Mean (SD)	IQR	Range	Mean (SD)	IQR	Range	
Angola, 2016	17,367	16,363	15.5 (8.7)	9.4	(5.7–42.5)	18.6 (5.1)	5.9	(10.8–36.2)	0.31
Burkina Faso, 2010	17,249	16,238	38.9 (23.6)	23.4	(14.8–147.5)	61.0 (5.6)	8.9	(48.4–72.6)	0.49
Burundi, 2017	16,949	16,497	21.2 (6.3)	8.3	(11.8–46.9)	24.4 (2.8)	3.3	(19.3–35.2)	0.27
Burundi, 2010	9403	9138	25.7 (7.5)	11.7	(10.1–42.8)	20.0 (3.0)	3.6	(15.2–30.6)	0.09
Cameroon, 2018	13,102	12,937	19.5 (9.7)	13.8	(6.0–65.7)	45.3 (8.8)	11.6	(24.5–70.2)	0.48
CDR, 2014	20,309	18,442	21.2 (15.4)	7.3	(7.1–70.1)	34.0 (7.4)	9.2	(14.5–51.3)	0.22
Ethiopia, 2016	18,324	17,720	23.0 (6.2)	6.6	(5.6–43.3)	22.1 (4.6)	4.3	(9.3–34.4)	0.53
Ethiopia, 2005	15,033	14,830	19.3 (7.1)	8.0	(4.7–42.6)	15.9 (3.9)	5.9	(6.0–25.3)	0.81
Ghana, 2014	12,934	12,180	19.5 (6.5)	7.1	(9.9–37.8)	46.1 (2.7)	3.7	(39.1–54.7)	0.23
Ghana, 2008	12,233	11,543	21.6 (7.5)	5.4	(12.3–58.9)	73.9 (6.2)	7.1	(58.5–86.9)	−0.04
Guinea, 2018	9170	8647	56.1 (4.9)	5.8	(43.4–77.4)	49.7 (3.6)	5.1	(43.0–60.5)	0.48
Kenya, 2014	39,985	38,935	10.1 (3.4)	5.2	(3.9–34.8)	13.1 (2.1)	2.9	(7.3–18.3)	0.45
Kenya, 2008	10,174	9871	8.9 (3.3)	3.7	(3.7–21.6)	8.9 (2.4)	3.9	(5.8–16.0)	0.67
Malawi, 2016	27,594	26,243	21.9 (8.4)	15.3	(8.2–42.3)	13.7 (1.1)	1.3	(11.4–17.4)	0.37
Malawi, 2010	27,516	26,381	6.4 (1.9)	1.2	(2.7–14.3)	11.9 (0.6)	0.8	(10.2–13.7)	−0.03
Mali, 2018	11,269	10,665	33.6 (11.3)	14.3	(13.5–74.5)	59.4 (3.6)	5.1	(52.6–68.5)	0.42
Mali, 2012	11,723	11,723	44.5 (15.0)	8.2	(20.9–91.9)	59.9 (4.3)	5.8	(50.4–72.4)	0.31
Mozambique, 2011	14,510	13,715	9.9 (6.2)	6.2	(3.5–35.6)	9.7 (2.8)	4.1	(4.4–16.9)	0.67
Nigeria, 2018	46,230	44,896	32.0 (12.7)	18.9	(11.4–104.5)	78.8 (9.9)	15.9	(47.0–101.0)	0.27
Rwanda, 2015	12,834	12,339	25.8 (9.5)	12.7	(12.4–46.6)	23.8 (2.1)	2.7	(17.4–28.6)	0.14
Rwanda, 2010	12,897	12,402	25.8 (9.4)	12.5	(12.4–46.6)	23.8 (2.1)	2.7	(17.4–28.6)	0.14
Senegal, 2014	7063	6715	49.2 (17.0)	25.3	(17.0–90.7)	42.3 (3.1)	4.6	(36.1–51.4)	0.04
Senegal, 2015	7286	6939	61.5 (27.5)	52.6	(16.6–112.9)	51.4 (4.6)	6.8	(39.2–61.7)	−0.13
Senegal, 2016	7147	6788	48.4 (26.1)	45.2	(16.8–107.1)	53.6 (4.4)	6.2	(42.7–64.3)	0.49
South Africa, 2016	13,792	13,712	11.2 (4.5)	7.5	(2.3–33.6)	11.2 (3.8)	6.0	(3.4–21.5)	0.94
Tanzania, 2016	14,449	13,549	13.2 (6.6)	5.6	(5.8–43.2)	13.6 (3.7)	4.9	(7.9–27.3)	0.73
Togo, 2014	10,384	10,204	64.6 (30.8)	54.3	(21.0–132.2)	52.7 (5.6)	9.7	(42.9–76.2)	−0.25
Uganda, 2016	21,356	20,112	19.8 (7.3)	7.6	(10.3–43.9)	24.9 (7.0)	11.7	(12.5–39.9)	0.71
Zambia, 2018	13,905	13,229	18.6 (11.8)	21.1	(4.0–52.4)	12.6 (2.1)	3.1	(8.7–20.1)	0.25
Zimbabwe, 2015	11,202	11,174	15.6 (7.2)	14.1	(5.2–35.9)	11.4 (1.4)	2.5	(8.8–15.6)	0.37
Zimbabwe, 2005	10,784	10,728	11.9 (7.5)	11.6	(3.3–35.8)	8.5 (0.9)	1.1	(6.7–12.4)	0.38

SD, standard deviation; IQR, interquartile range. * Correlation ratio between the month of PM_2.5_ exposure preceding the survey interview and the annual PM_2.5_ exposure.

**Table 3:** Associations between prior-month PM_2.5_ exposure (per 1 μg/m^3^ higher) and prevalence of cough or ALRI in the past two weeks across Demographic and Health Surveys (DHS) in sub-Saharan Africa.

Variable	Cough				ALRI			
	*N*	OR [95%CI]	*I*^2^ (%)	*P*_het_	*N*	OR [95%CI]	*I*^2^ (%)	*P*_het_
All	368,366	1.000 [0.981, 1.009]	50.4	0.001	109,644	0.975 [0.941, 1.010]	67.4	<0.001
Cluster type								
Urban	111,729	0.999 [0.980, 1.018]	71.3	<0.001	30,582	0.971 [0.910, 1.037]	62.5	0.001
Rural	256,637	0.999 [0.987,1.011]	67.9	<0.001	76,612	0.975 [0.957,0.993]	43.6	0.053
Wealth index								
Q1–Q2	93,114	1.006 [0.987, 1.024]	65.0	<0.001	23,461	0.940 [0.913, 0.969]	16.1	0.291
Q3–Q5	101,236	0.991 [0.975, 1.008]	59.1	<0.001	23,611	0.974 [0.939, 1.011]	48.7	0.034
Stunting								
Stunted	44,197	0.991 [0.972, 1.012]	40.0	0.015	9249	0.966 [0.851, 1.096]	79.1	<0.001
Non-stunted	280,787	1.001 [0.992, 1.011]	45.8	0.003	73,734	0.993 [0.963, 1.025]	57.6	0.009
Sex								
Boys	159,179	1.000 [0.988, 1.013]	53.0	<0.001	38,660	0.983 [0.934, 1.036]	58.2	0.008
Girls	156,223	1.001 [0.993, 1.008]	26.9	0.086	36,517	0.976 [0.942, 1.010]	12.6	0.327
Age								
<24 months	60,622	0.996 [0.964, 1.029]	54.4	0.010	32,020	0.976 [0.935, 1.018]	46.1	0.046
24–59 months	88,685	0.993 [0.977, 1.010]	4.4	0.403	43,220	0.987 [0.947, 1.029]	54.3	0.016
Location								
West Africa	115,221	1.002 [0.989, 1.015]	65.4	0.001	30,493	0.945 [0.861, 1.037]	80.6	0.001
Rest of Africa	253,145	0.996 [0.989, 1.004]	33.0	0.082	79,151	0.983 [0.946, 1.021]	59.4	0.012
HDI index								
Medium-to-high	81,517	1.022 [0.982, 1.064]	63.3	0.018	17,201	1.018 [0.975, 1.064]	0	0.609
low	286,849	0.998 [0.989, 1.006]	45.4	0.008	92,443	0.958 [0.915, 1.002]	75.3	<0.001

Odds ratios were adjusted for a priori defined covariates, including child’s sex, child’s age in months, size at birth, month of the interview, household wealth index, maternal age, and maternal education. Wealth index Q1–Q2: quintile 1 to 2, corresponding to poorer households; Q3–Q5: quintile 3 to 5, corresponding to average or wealthier households. West Africa: Burkina Faso, Cameroon, Ghana, Guinea, Mali, Nigeria, Senegal and Togo. HDI index: medium-to-high (≥0.55), low (<0.55). *P*_het_: *p*-value for heterogeneity across surveys.

## Data Availability

The survey datasets analysed for the current study was provided by Demographic Health Surveys, and is publicly available upon reasonable request and with permission of DHS. Air quality data are publicly available.

## Supplementary Materials

The following are available online at https://www.mdpi.com/article/10.3390/ijerph18189729/s1, Text S1: Creation of the monthly 0.01-degree resolution grids for PM_2.5_. Table S1: Results of sensitivity analyses to the main model. Figures S1–S29: Associations between short-term PM_2.5_ exposure and odds of self-reported ALRI and cough.
