# Supplementary material for: Ambient Air Pollution and Respiratory Health in Sub-Saharan African Children: A Cross-Sectional Analysis
Source: Int J Environ Res Public Health. 2021 Sep 15;18(18):9729. doi: 10.3390/ijerph18189729 (PMC8467583; doi:10.3390/ijerph18189729)
Supplement: Supplementary file 1 [file ijerph-18-09729-s001.zip › ijerph-1377364-supplementary.pdf]

International Journal of
Environmental Research and Public Health

MDPI

Supplementary Material

Supplementary Text S1: Creation of the monthly 0.01-degree resolution grids for $PM_{2.5}$

These grids were generated by synthesising annual 0.01-degree resolution grids with monthly 0.625-degree resolution grids, following the method described in Xue et al [1].

In summary, annual $PM_{2.5}$ grids (v4.GL.03) were downloaded from the Atmospheric Composition Analysis Group (ACAG) (available online: http://fizz.phys.dal.ca/~atmos/martin/?page_id=140; accessed on 13 September 2021), and MERRA-2 data (available online: https://disc.gsfc.nasa.gov/SSW/#keywords=M2TMNXAER; accessed on 13 September 2021) were downloaded using the GES-DISC Simple Subset Wizard tool to select the required data bands before converting them to $PM_{2.5}$ estimates using:

$$\mathrm{PM_{2.5}MERRA} - 2 = 1.375 \times \mathrm{SO_4} + 1.8 \times \mathrm{OC} + \mathrm{BC} + \mathrm{DS_{2.5}} + \mathrm{SS_{2.5}}$$

The synthesised grids were then created using the formula:

$$\mathrm{HiRes}(y, m) = \mathrm{LowRes}(y, m) \times \mathrm{HiRes}(y)/\mathrm{mean}(\mathrm{LowRes}(y, 1)..\mathrm{LowRes}(y, 12))$$

where HiRes refer to the 0.01-degree ACAG data and LowRes to the 0.625-degree MERRA-2 data.

To implement this efficiently, we used the following algorithm:

- For each year:
    - Read the annual high-resolution ACAG raster HiRes(y)
    - Create and cache an annual average of the 12 MERRA-2 rasters, at the native 0.625-degree resolution, converting to the same units used in the ACAG data
    - Read the (cached) annual average MERRA-2 raster, upsampling it on read using bilinear interpolation to the extent and shape matching the ACAG raster (arrays will be of same dimensions), sum(LowRes(y,*1*)..LowRes(y,*12*)). Note that combining the grids using a standard GIS tool would implicitly perform a nearest-neighbour interpolation of the 0.625-degree data, leading to an apparenty blockiness in the synthesised output data.
    - Calculate the correction factor for this year HiRes(y) / sum(LowRes(y,*1*)..LowRes(y,*12*))
    - For each month in the year:
        - Read the month's MERRA-2 raster, upsampling it in the same way as above LowRes(y,m), converting to the same units as the ACAG-2 and MERRA-2 annuals
        - Multiply it by the year's correction factor raster
        - Mask where either the ACAG or MERRA rasters were masked

The python implementation can be found in this notebook (available online: https://github.com/harry-gibson/geo_notebooks/blob/master/upscale_raster_from_other_dataset.ipynb; accessed on 13 September 2021).

Extraction of the $PM_{2.5}$ data to survey locations

We extracted a series of 12 monthly $PM_{2.5}$ values from these synthesised $PM_{2.5}$ rasters for every DHS household, based on their (aggregated) cluster latitude/longitude and the actual date of the each household's interview.

That is, although multiple households are recorded at the same lat/lon, if they were interviewed in different months then a different set of rasters were extracted for the 12 months prior to interview.

The 15th of the month was used as the reference date for allocation of household to reference month. For example, any household interviewed between 15 July 2001 and 14 August 2001 (inclusive) would be counted as "July 2001" and would receive data from July 2001 for month 0, June 2001 for month –1, etc, through to August 2000 for month –12.

The rationale for this was that the questions relating to health outcomes generally relate to the last two weeks (e.g. did a child suffer from a cough in the last two weeks).

References:

1. Xue, T., Zhu, T., Geng, G. & Zhang, Q. Association between pregnancy loss and ambient $PM_{2.5}$ using survey data in Africa: A longitudinal case-control study, 1998–2016. *Lancet Planet. Heal.* **2019**, *3*, e219–ee225.

Table S1. Results of sensitivity analyses to the main model.

Model	Cough			ALRI		
	**OR (95%CI)**	**I^2 (%)**	**P_{het}**	**OR (95%CI)**	**I^2 (%)**	**P_{het}**
Main model	1.000 (0.981-1.009)	50.4	0.001	0.975 (0.941-1.010)	67.4	<0.001
Main model+ health card ownership	1.001 (0.992-1.009)	37.6	0.021	0.985 (0.952-1.019)	50.9	0.021
Main model+ types of cooking fuel	0.999 (0.990-1.007)	47.7	0.002	0.971 (0.937-1.006)	66.9	<0.001
Main model+ child stunting status	0.999 (0.990-1.007)	47.1	0.002	0.974 (0.938-1.011)	66.5	<0.001

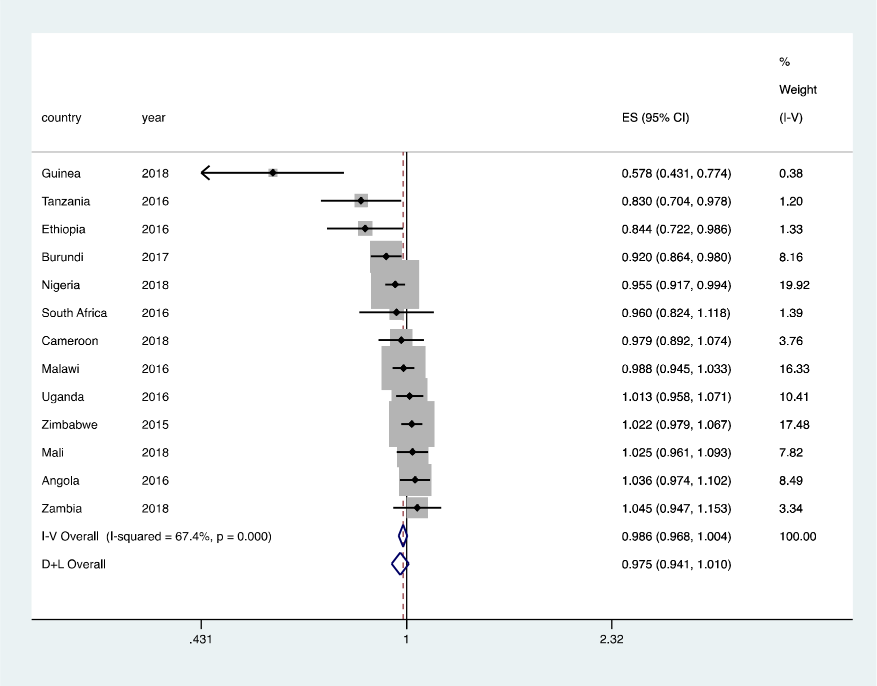

Figure S1. Associations between short-term $PM_{2.5}$ concentration and odds of self-reported ALRI.

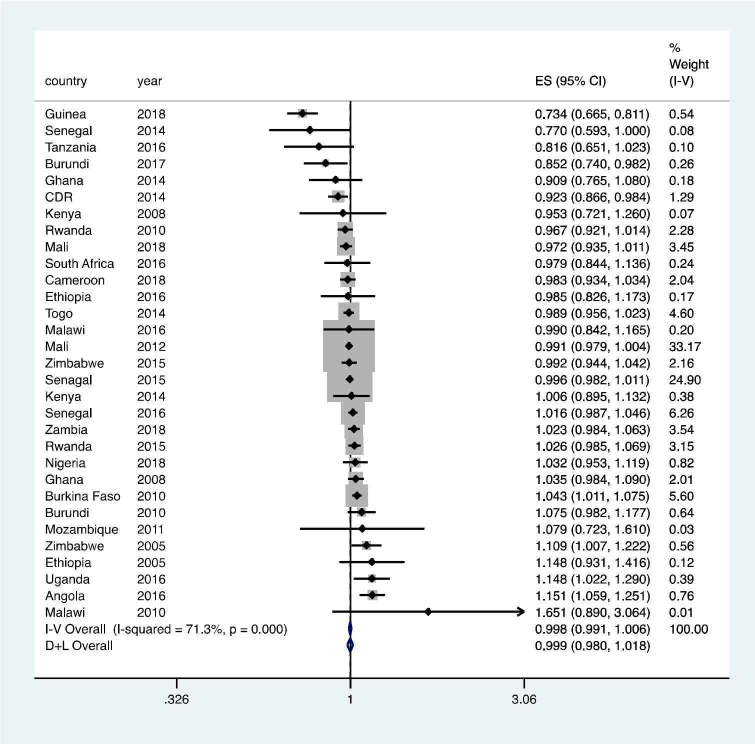

Figure S2. Associations between short-term $PM_{2.5}$ concentration and odds of self-reported cough among urban clusters.

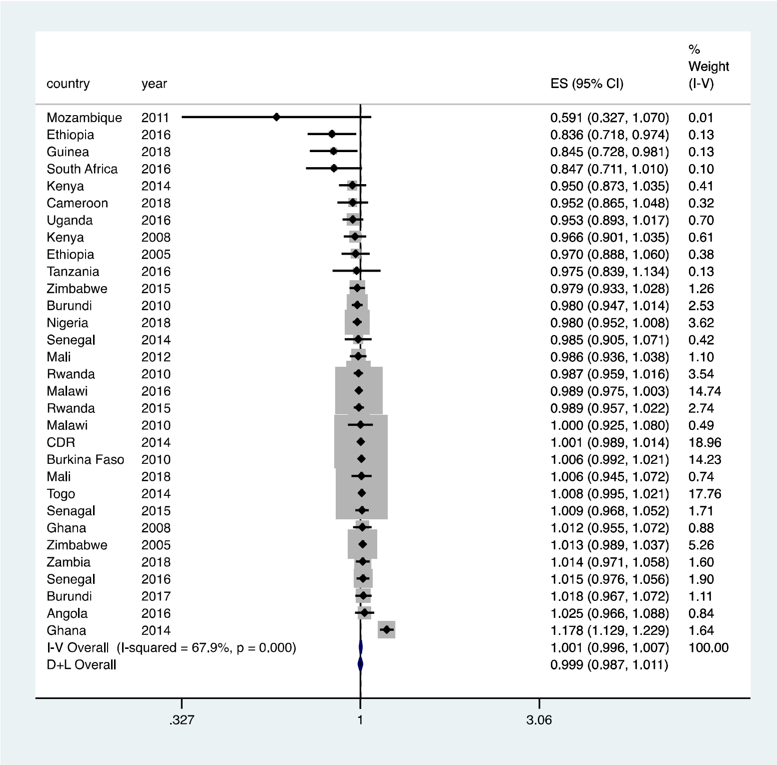

Figure S3. Associations between short-term $PM_{2.5}$ concentration and odds of self-reported cough among rural clusters.

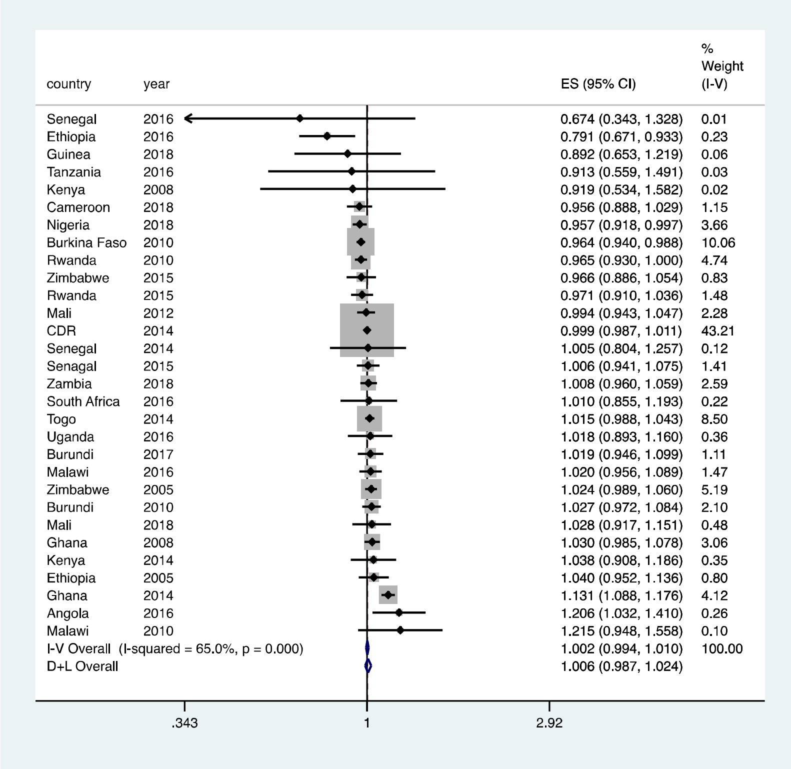

Figure S4. Associations between short-term PM2.5 concentration and odds of self-reported cough among Q1–Q2 of household wealth index.

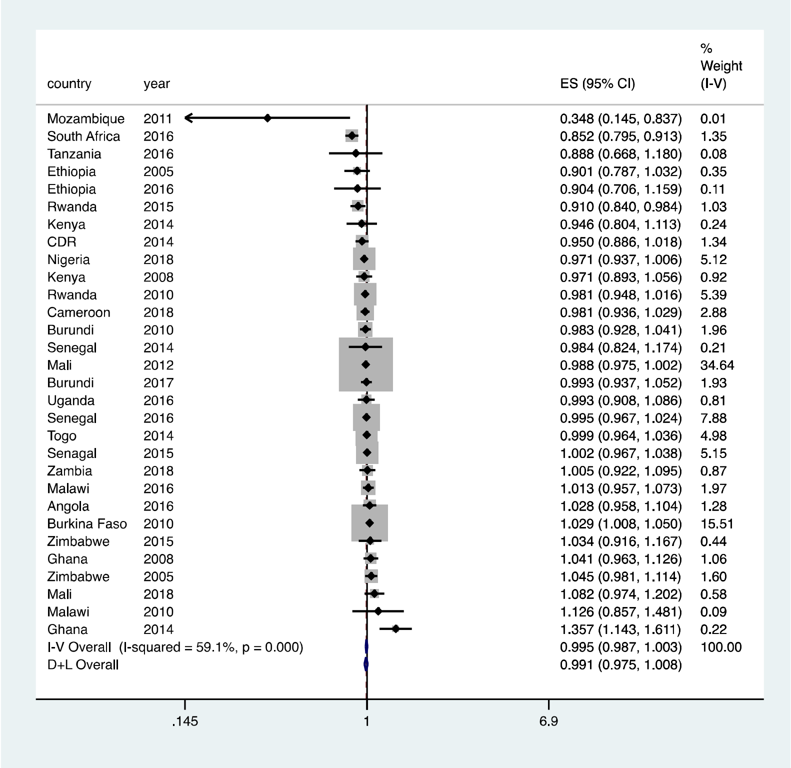

Figure S5. Associations between short-term PM2.5 concentration and odds of self-reported cough among Q3–Q5 of household wealth index.

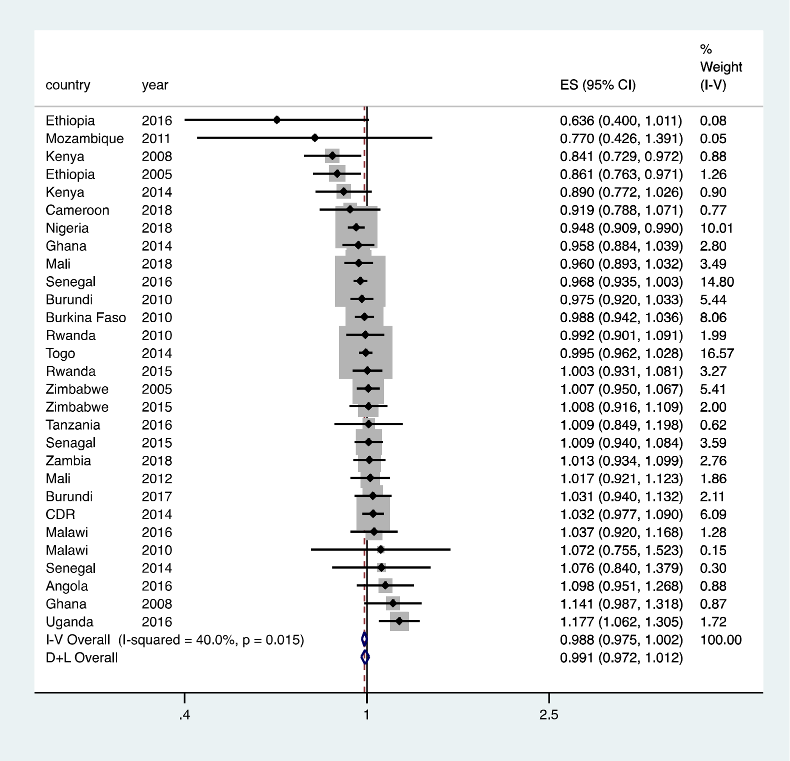

Figure S6. Associations between short-term $PM_{2.5}$ concentration and odds of self-reported cough among stunting children.

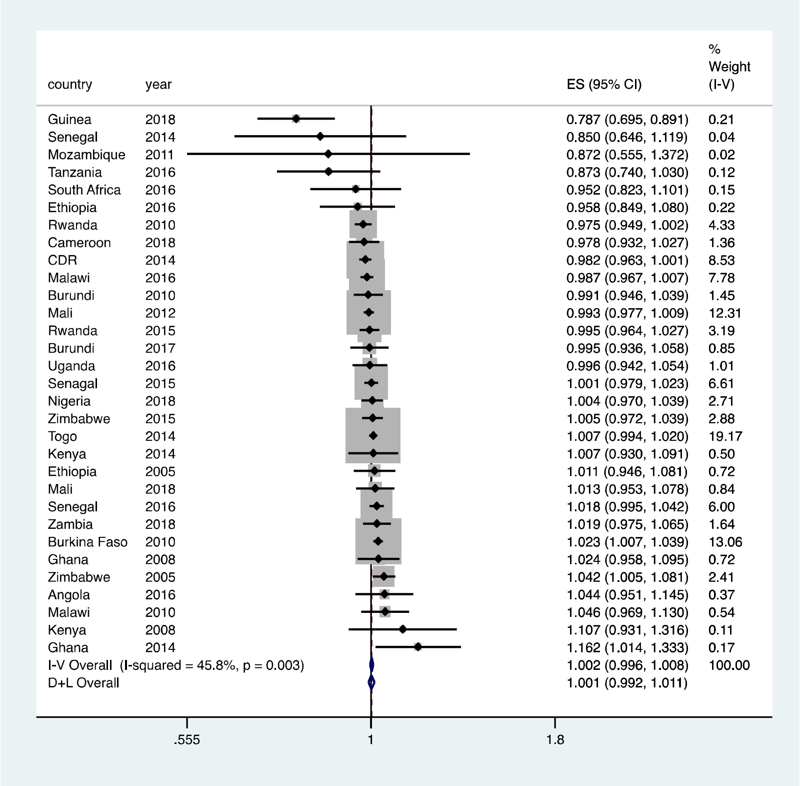

Figure S7. Associations between short-term $PM_{2.5}$ concentration and odds of self-reported cough among non-stunting children.

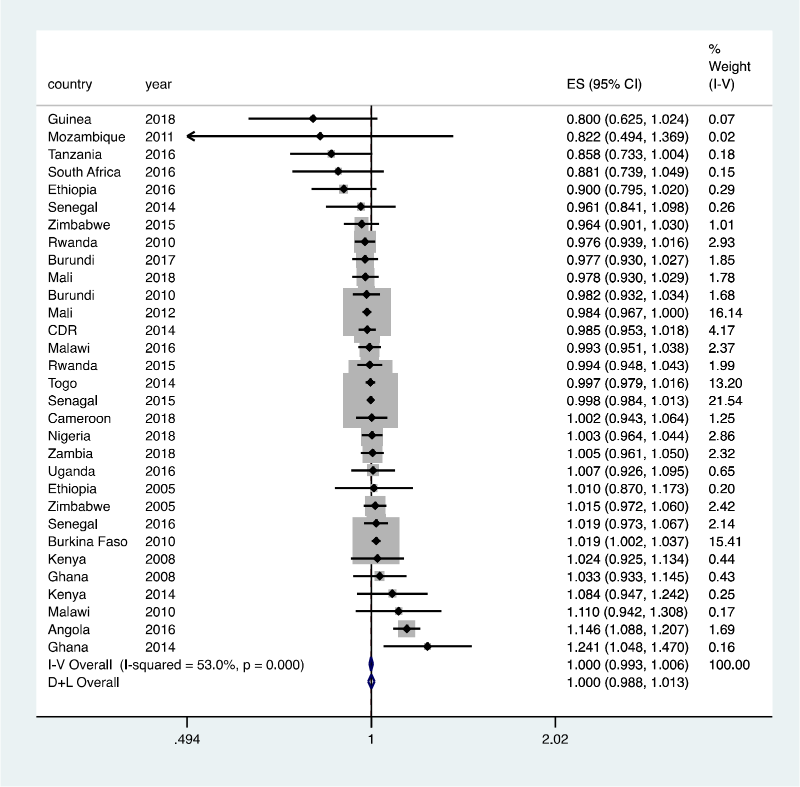

Figure S8. Associations between short-term $PM_{2.5}$ concentration and odds of self-reported cough among boys.

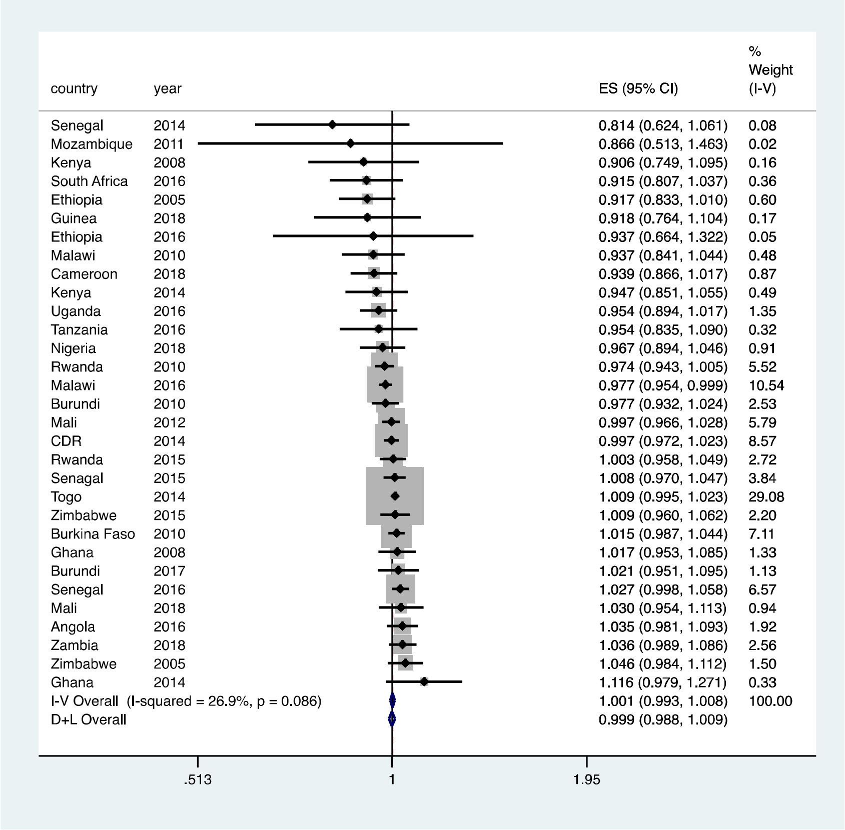

Figure S9. Associations between short-term $PM_{2.5}$ concentration and odds of self-reported cough among girls.

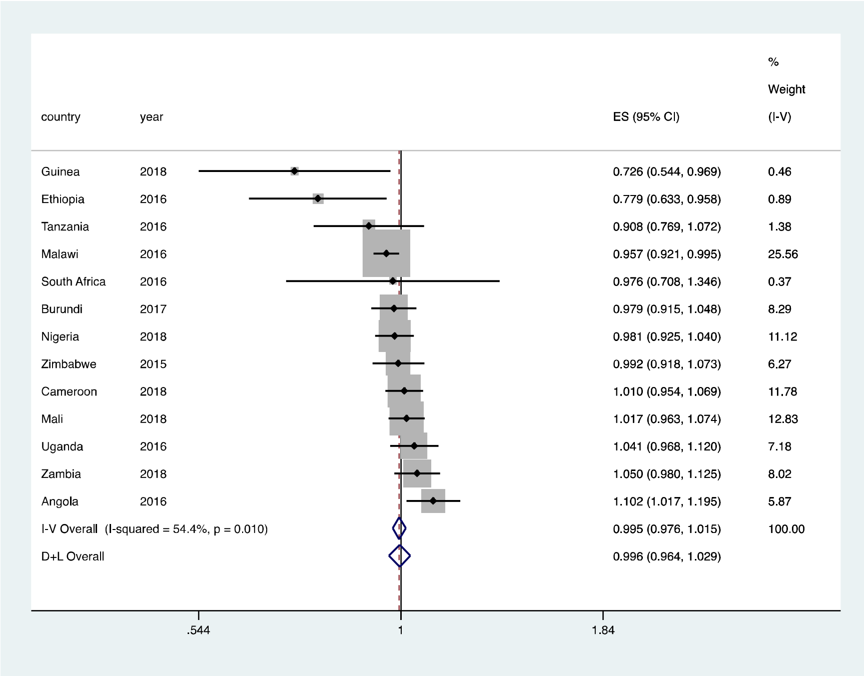

Figure S10. Associations between short-term $PM_{2.5}$ concentration and odds of self-reported cough among children under 2 years of age.

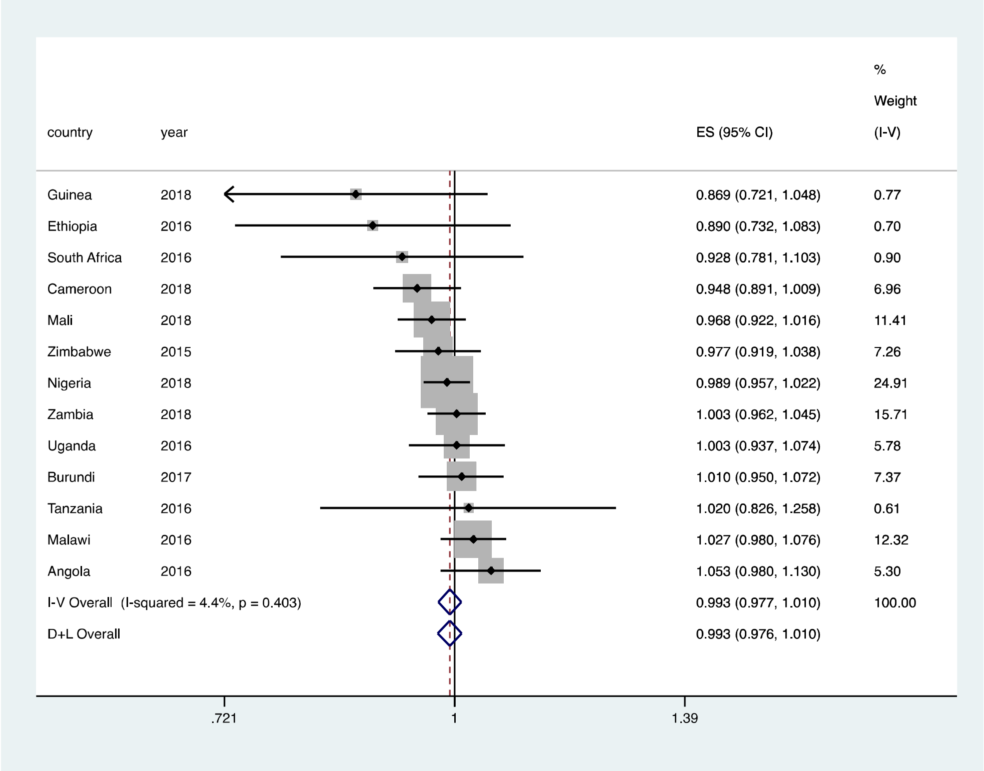

Figure S11. Associations between short-term $PM_{2.5}$ concentration and odds of self-reported cough among children 2–5 years of age.

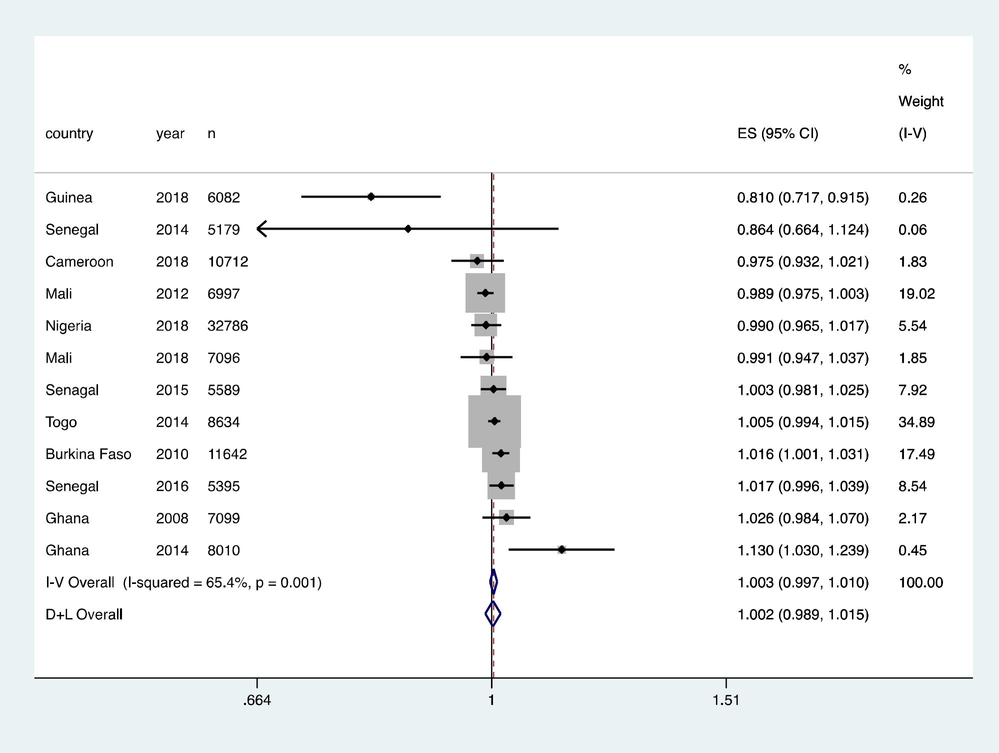

Figure S12. Associations between short-term PM2.5 concentration and odds of self-reported cough among west sub-Saharan African countries.

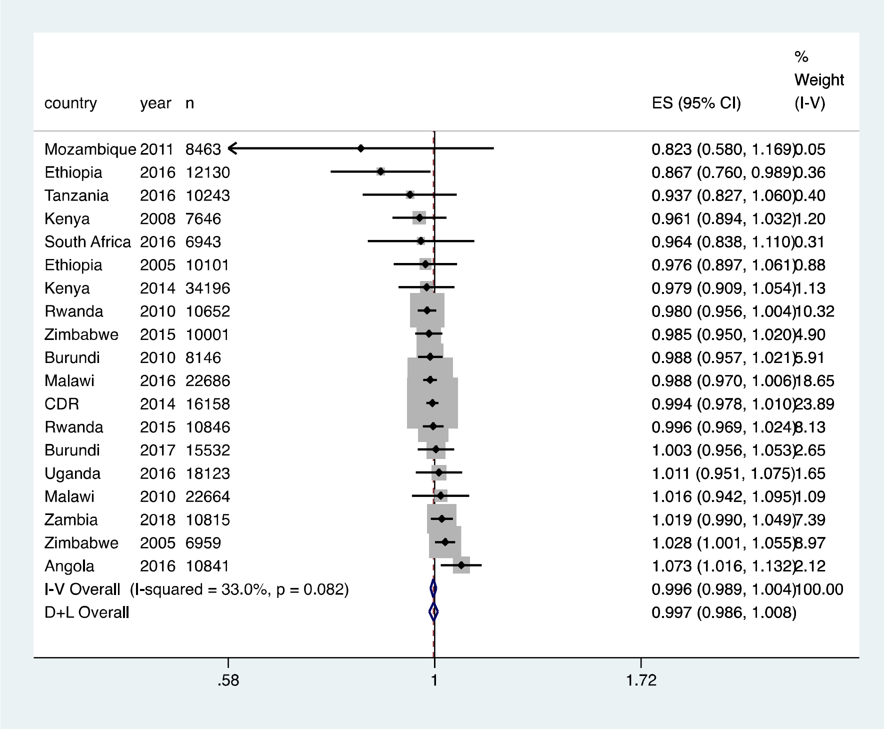

Figure S13. Associations between short-term PM2.5 concentration and odds of self-reported cough among rest of African countries.

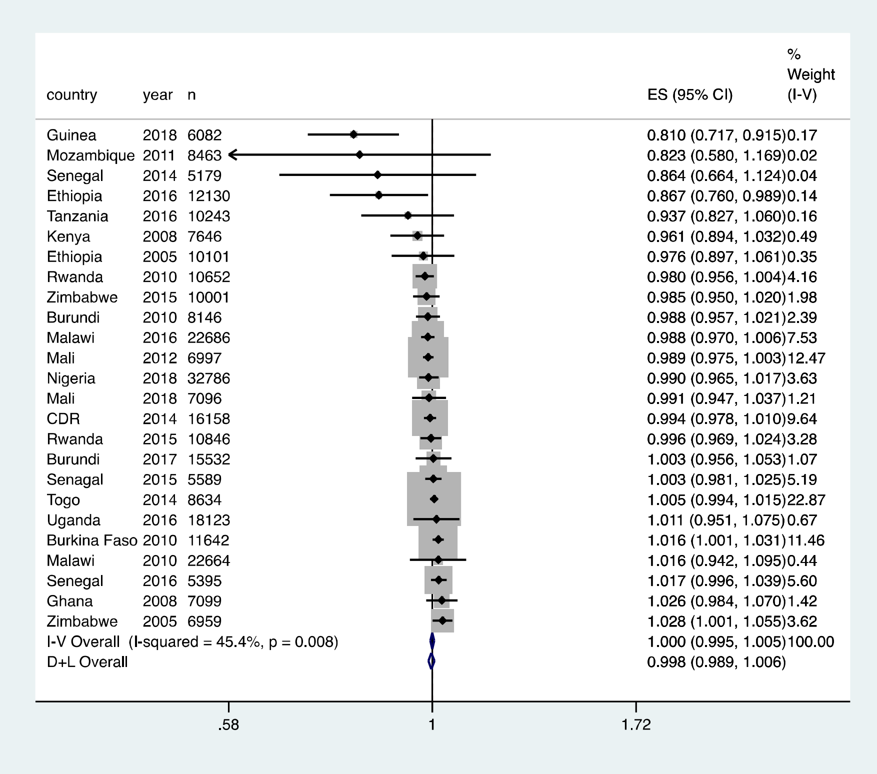

Figure S14. Associations between short-term $PM_{2.5}$ concentration and odds of self-reported cough among countries with a low HDI (<0.55).

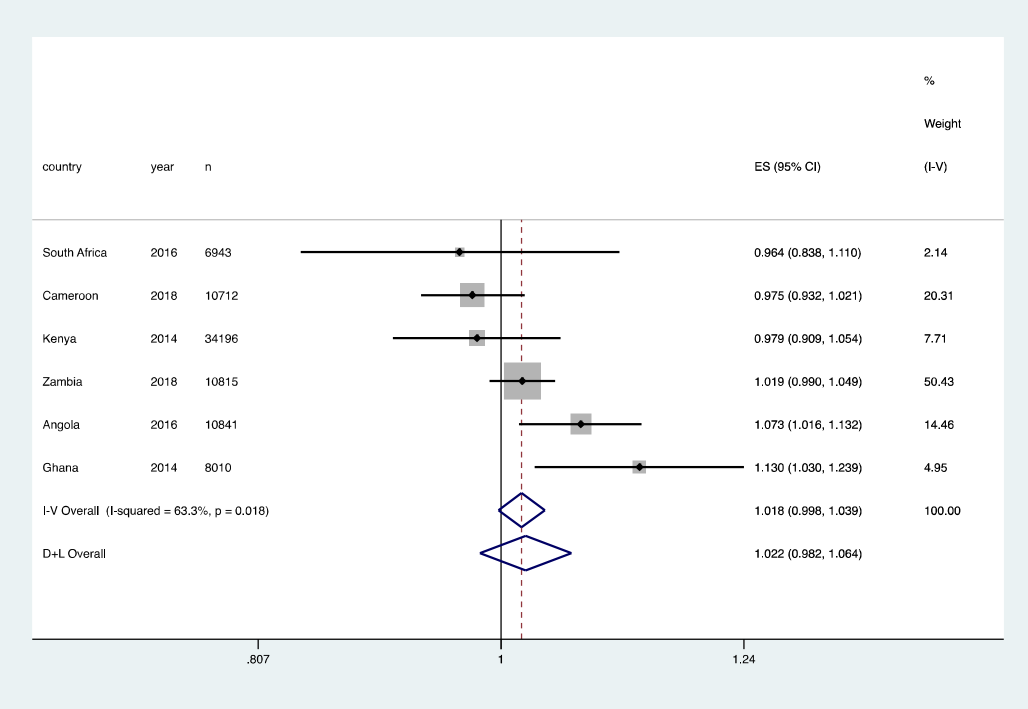

Figure S15. Associations between short-term $PM_{2.5}$ concentration and odds of self-reported cough among countries with a medium-to-high HDI (≥0.55).

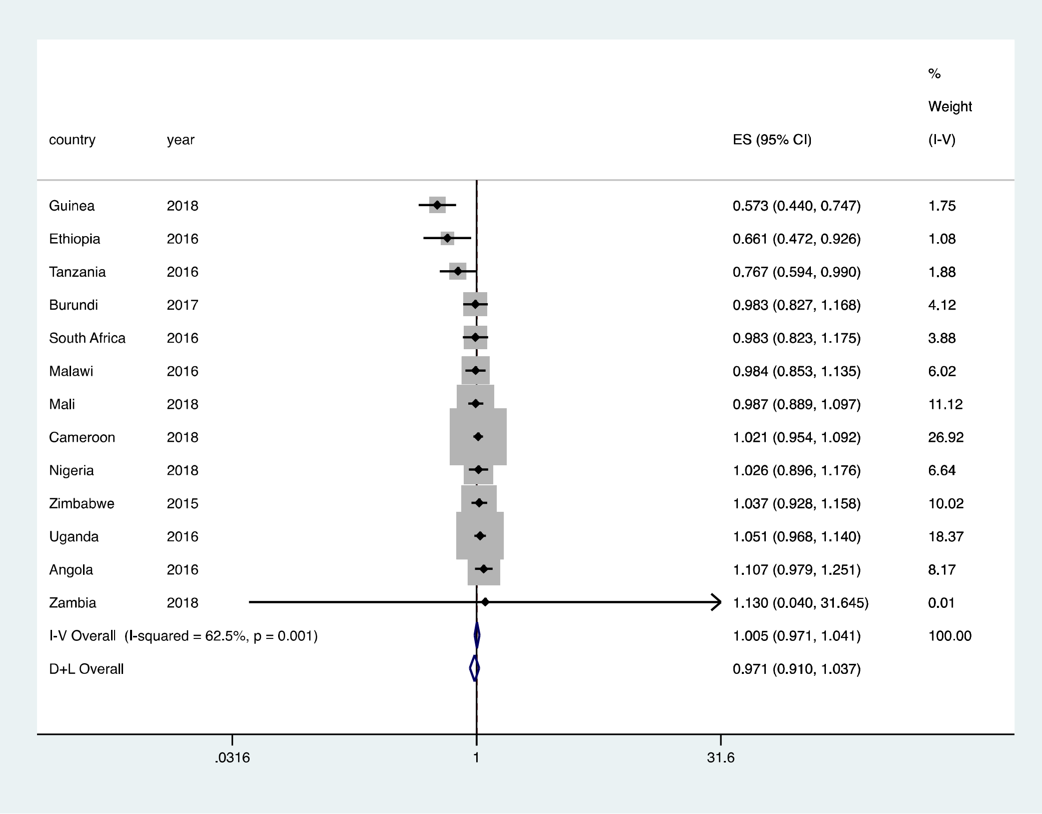

Figure S16. Associations between short-term PM2.5 concentration and odds of self-reported ALRI among urban clusters.

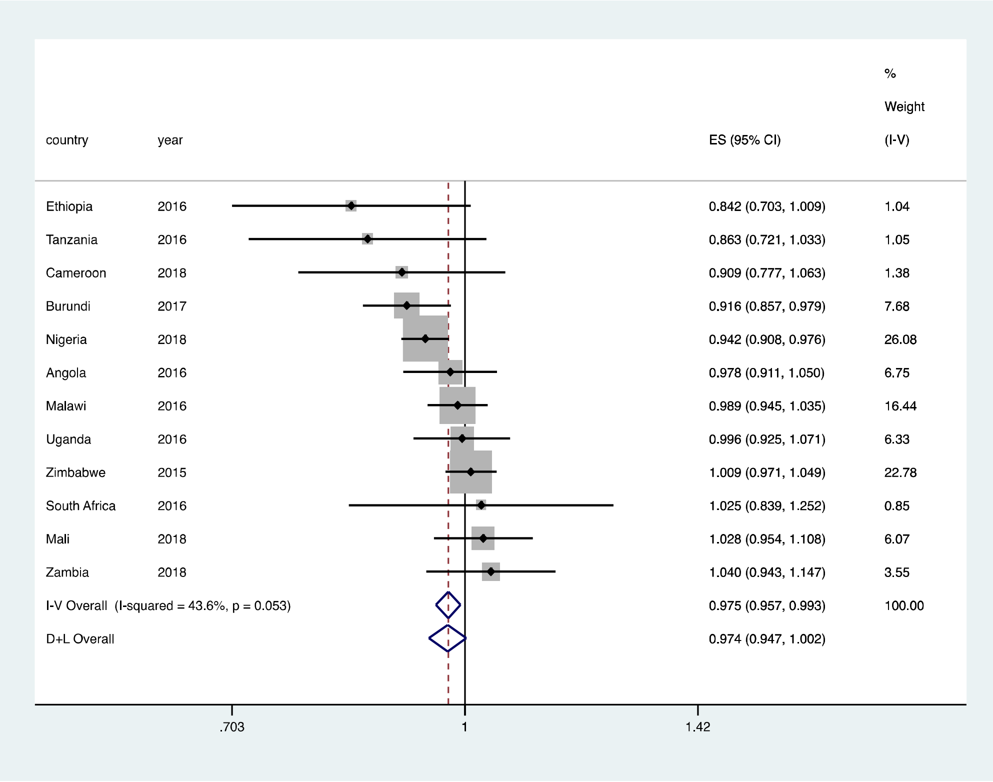

Figure S17. Associations between short-term PM2.5 concentration and odds of self-reported ALRI among rural clusters.

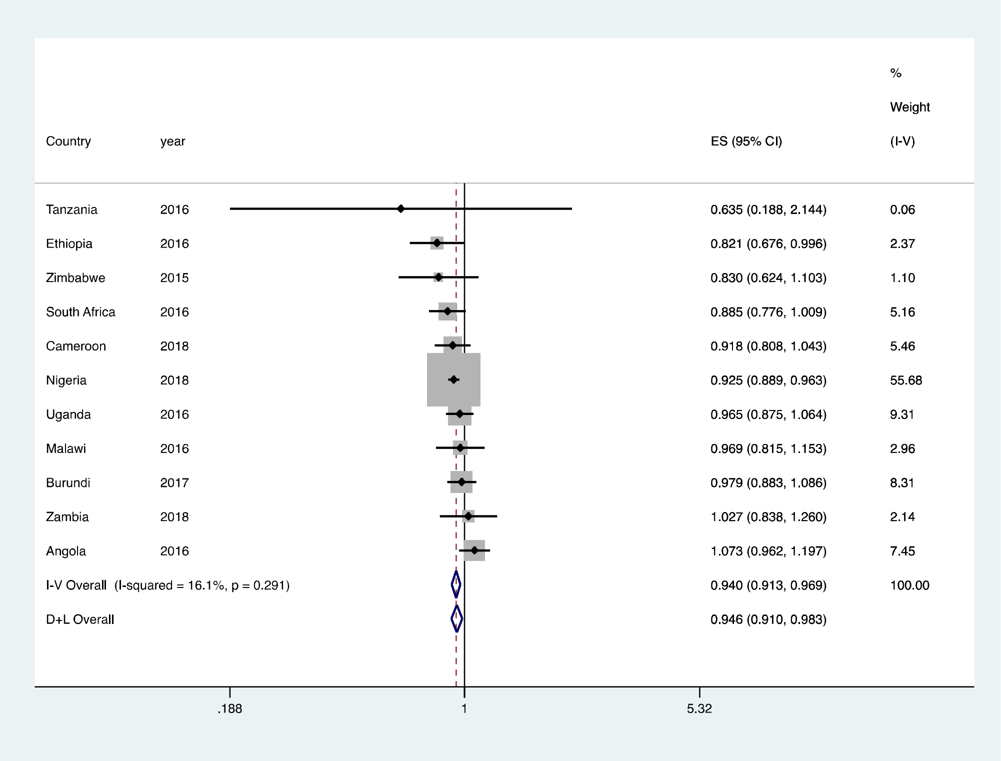

Figure S18. Associations between short-term $PM_{2.5}$ concentration and odds of self-reported ALRI among Q1–Q2 household wealth index .

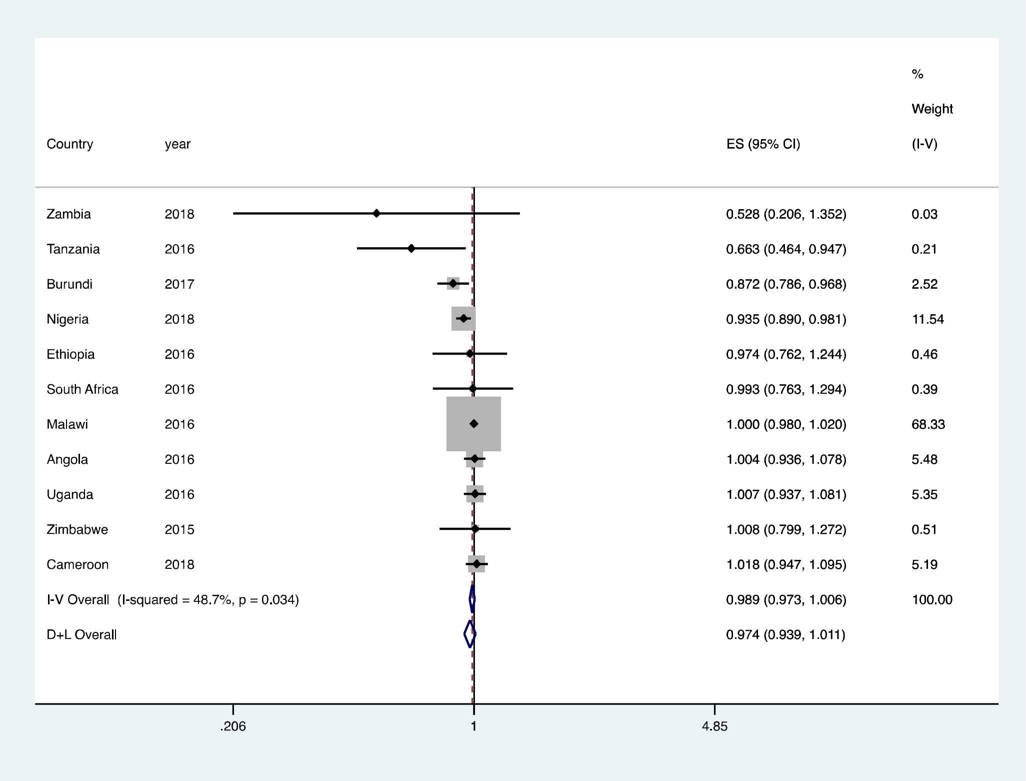

Figure S19. Associations between short-term $PM_{2.5}$ concentration and odds of self-reported ALRI among Q3–Q5 household wealth index .

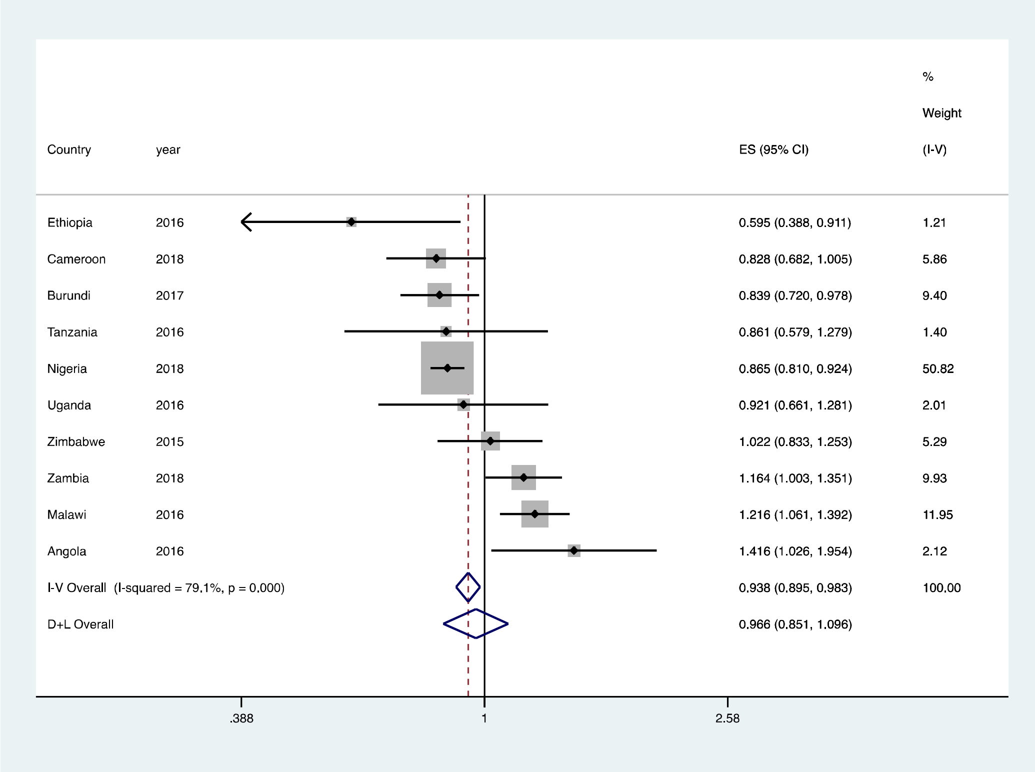

Figure S20. Associations between short-term PM2.5 concentration and odds of self-reported ALRI among stunted children.

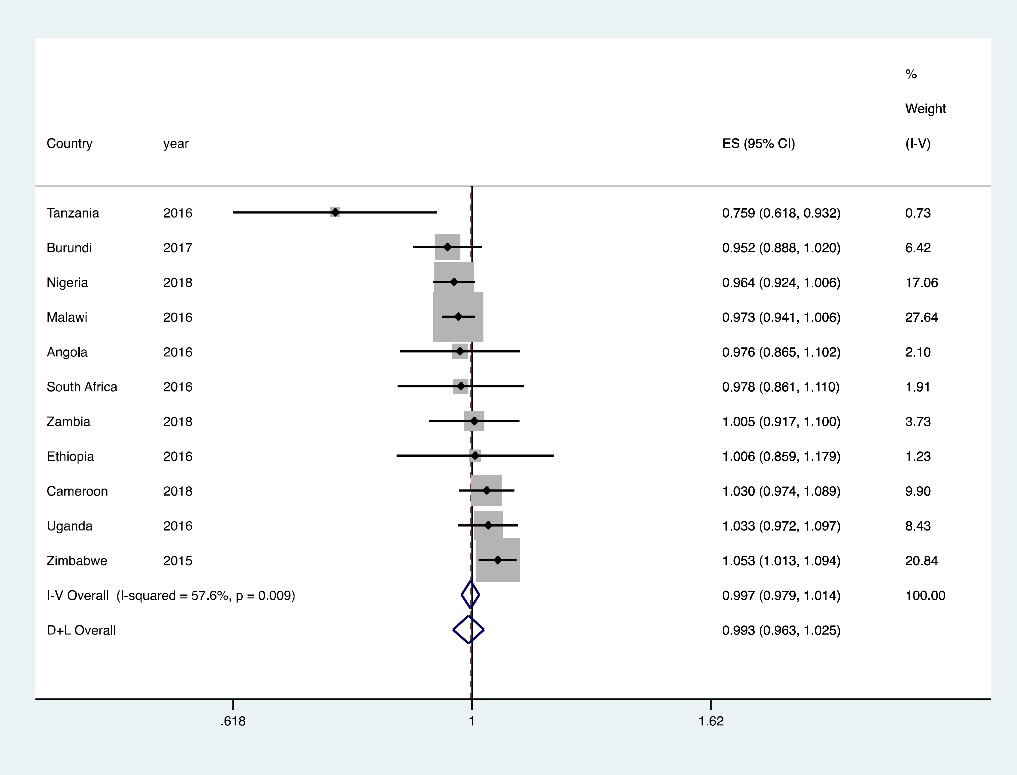

Figure S21. Associations between short-term PM2.5 concentration and odds of self-reported ALRI among non-stunted children.

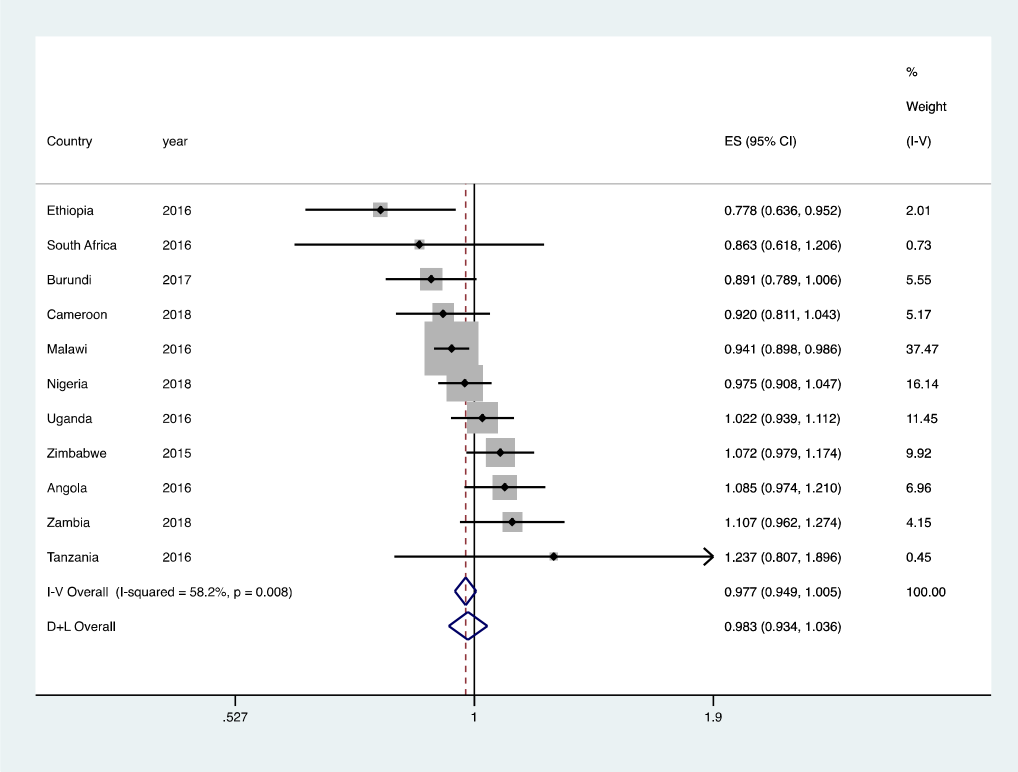

Figure S22. Associations between short-term $PM_{2.5}$ concentration and odds of self-reported ALRI among boys.

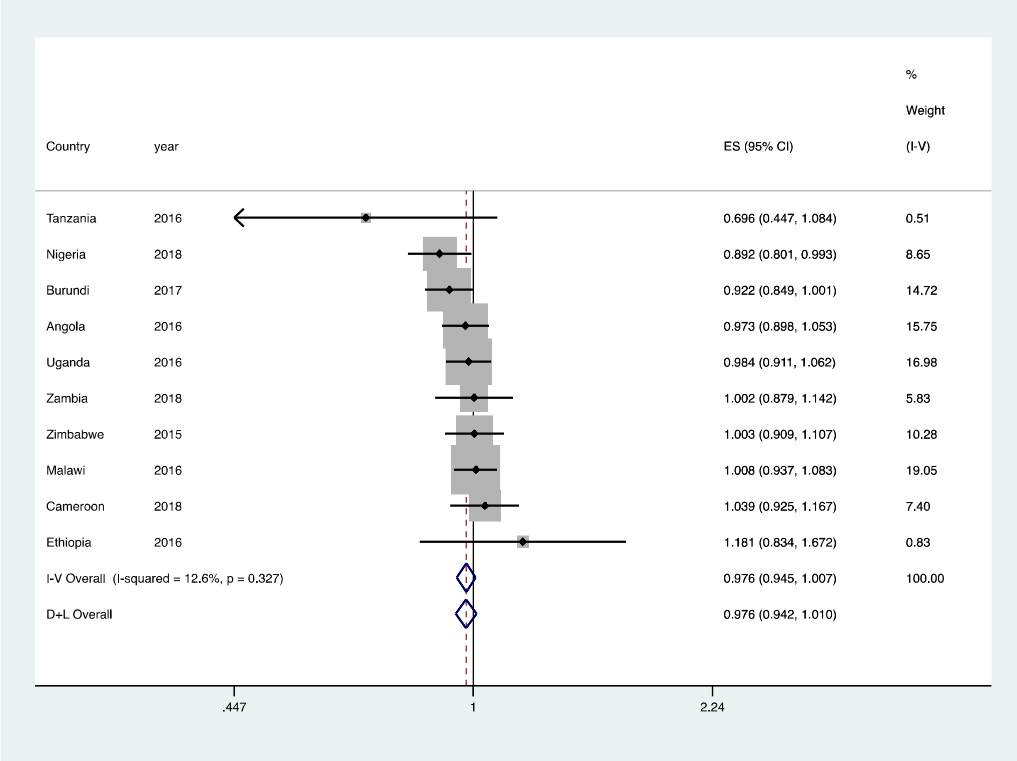

Figure S23. Associations between short-term $PM_{2.5}$ concentration and odds of self-reported ALRI among girls.

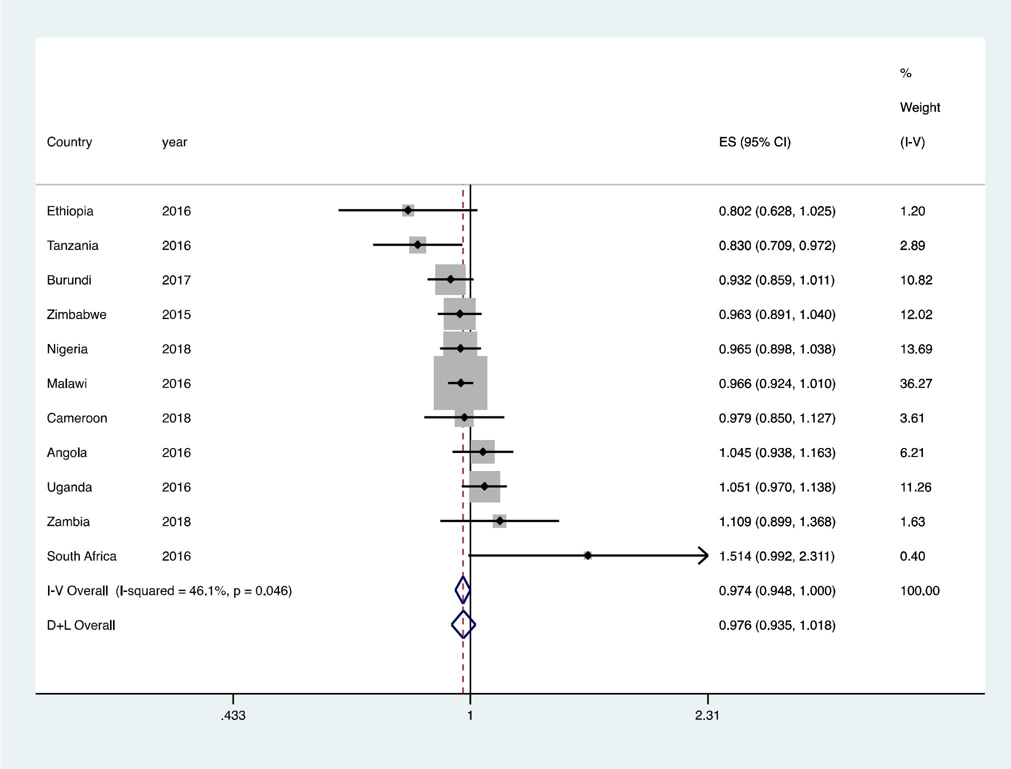

Figure S24. Associations between short-term $PM_{2.5}$ concentration and odds of self-reported ALRI among children under 2 years of age.

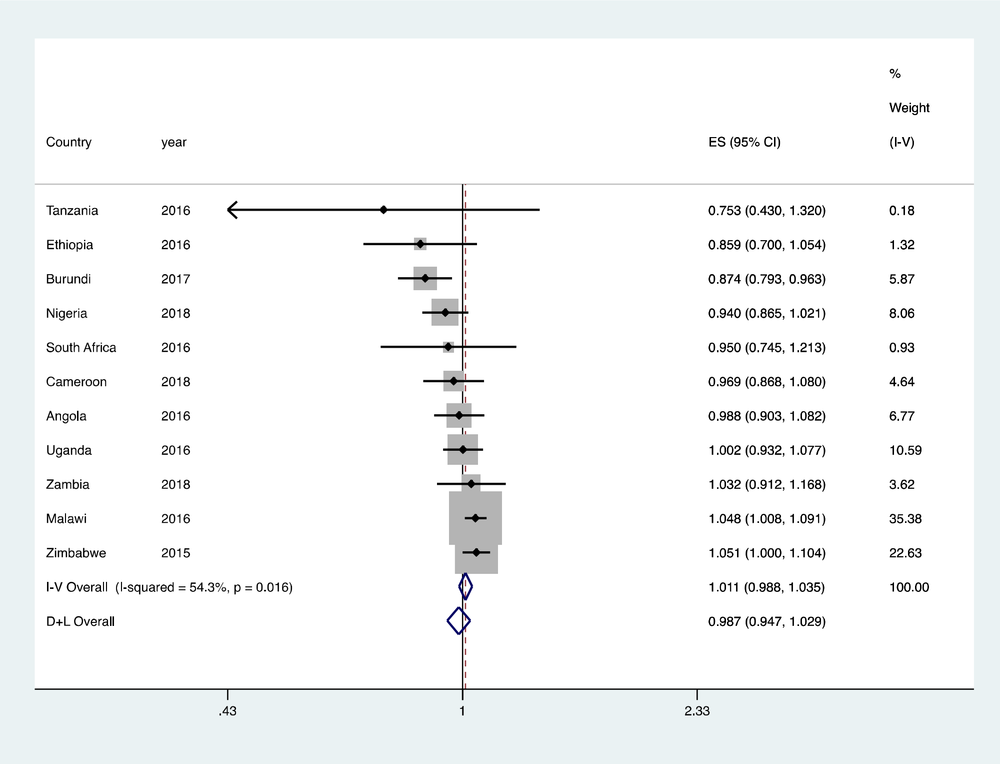

Figure S25. Associations between short-term $PM_{2.5}$ concentration and odds of self-reported ALRI among children between 2 and 5 years of age.

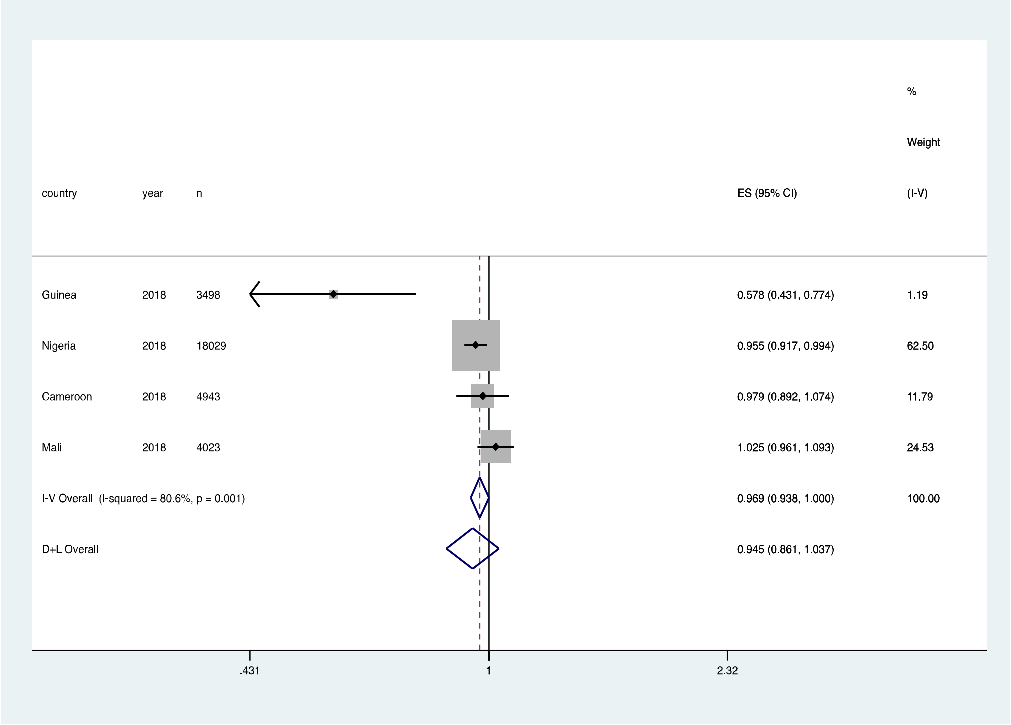

Figure S26. Associations between short-term $PM_{2.5}$ concentration and odds of self-reported ALRI among west sub-Saharan African countries.

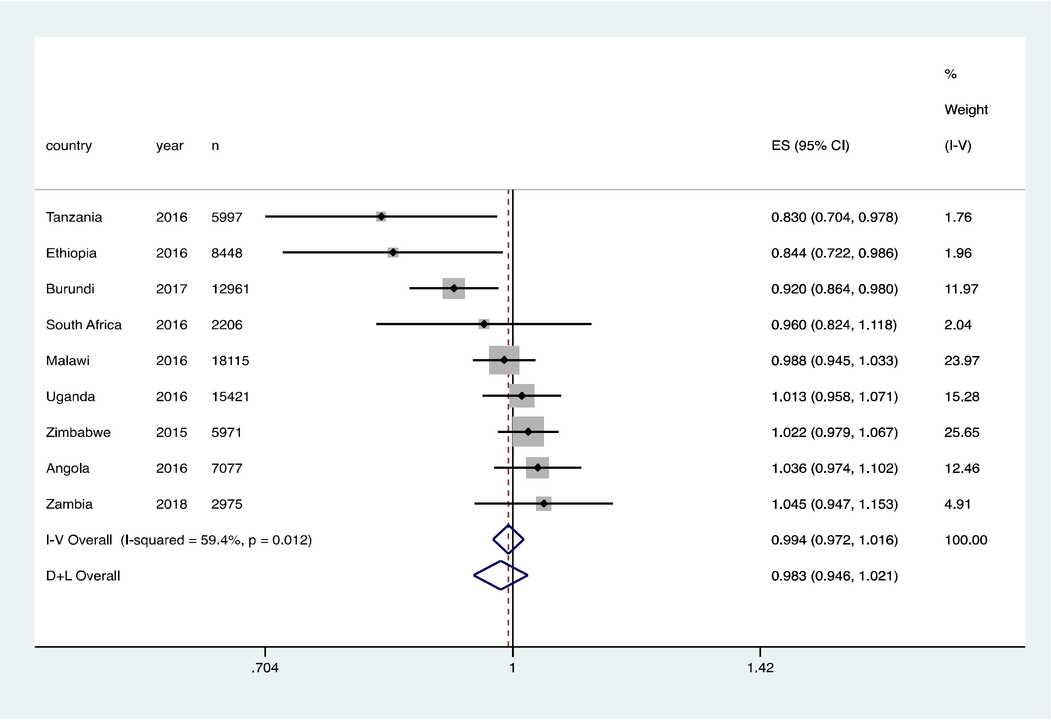

Figure S27. Associations between short-term $PM_{2.5}$ concentration and odds of self-reported ALRI among the rest of sub-Saharan African countries.

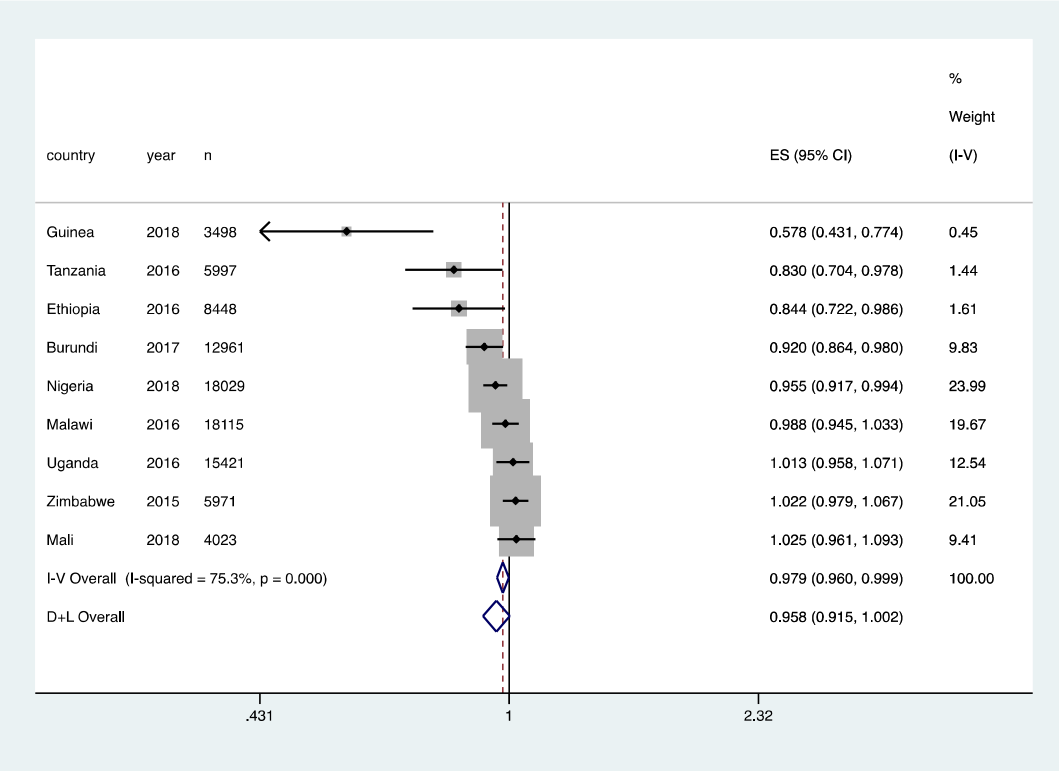

Figure S28. Associations between short-term $PM_{2.5}$ concentration and odds of self-reported ALRI among countries with a low HDI (<0.55).

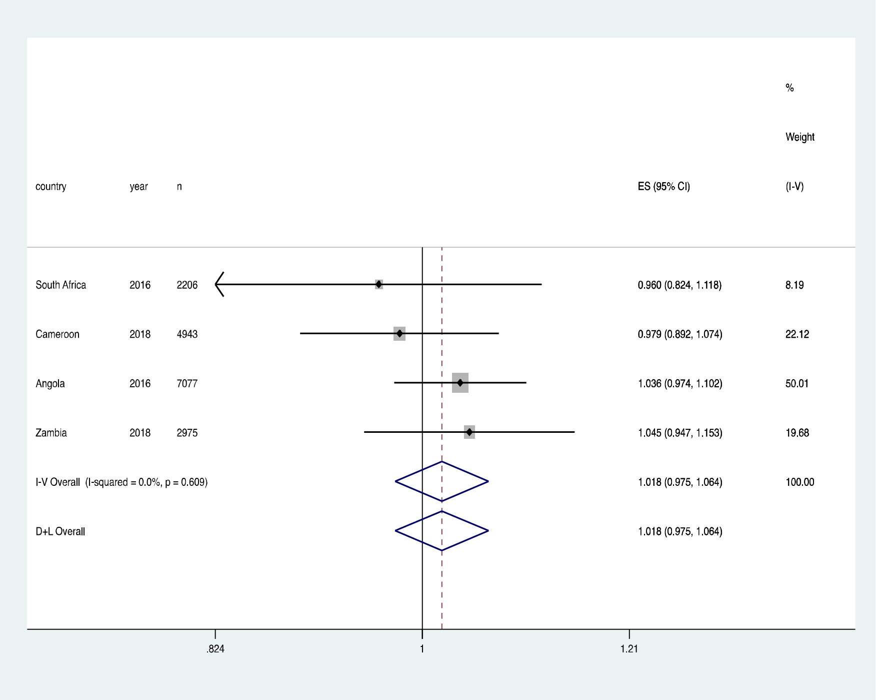

Figure S29. Associations between short-term $PM_{2.5}$ concentration and odds of self-reported ALRI among countries with a medium-to-high HDI (≥0.55).
